# The jumping spiders from Xishuangbanna, Yunnan, China (Araneae, Salticidae)

**DOI:** 10.3897/zookeys.630.8466

**Published:** 2016-11-09

**Authors:** Qi Cao, Shuqiang Li, Marek Żabka

**Affiliations:** 1 Institute of Zoology, Chinese Academy of Sciences, Beijing 100101, China; 2 University of the Chinese Academy of Sciences, Beijing 100049, China; 3 Southeast Asia Biodiversity Research Institute, Chinese Academy of Sciences, Menglun, Mengla, Yunnan 666303, China; 4 Department of Zoology, University of Natural Sciences and Humanities, 08-110 Siedlce, Poland

**Keywords:** Description, diagnosis, new species, Southeast Asia, taxonomy

## Abstract

Twenty one jumping spider species from South Yunnan are reported, diagnosed, described and illustrated; 19 of them are described as new: *Afraflacilla ballarini* Cao & Li, **sp. n.** (♂), *Agorius tortilis* Cao & Li, **sp. n.** (♂♀), *Bavia exilis* Cao & Li, **sp. n.** (♂), *Carrhotus kevinlii* Cao & Li, **sp. n.** (♂♀), *Carrhotus sarahcrewsae* Cao & Li, **sp. n.** (♂), *Chinattus wengnanensis* Cao & Li, **sp. n.** (♂♀), *Chinophrys mengyangensis* Cao & Li, **sp. n.** (♂♀), *Cocalus menglaensis* Cao & Li, **sp. n.** (♂♀), *Cosmophasis xiaolonghaensis* Cao & Li, **sp. n.** (♂♀), *Cytaea yunnanensis* Cao & Li, **sp. n.** (♂), *Gedea pinguis* Cao & Li, **sp. n.** (♂), *Gelotia zhengi* Cao & Li, **sp. n.** (♂), *Icius bamboo* Cao & Li, **sp. n.** (♂), *Nannenus menghaiensis* Cao & Li, **sp. n.** (♂♀), *Pancorius latus* Cao & Li, **sp. n.** (♂), *Phintella lepidus* Cao & Li, **sp. n.** (♂♀), *Phintella sancha* Cao & Li, **sp. n.** (♂), *Ptocasius paraweyersi* Cao & Li, **sp. n.** (♂♀), and *Stenaelurillus fuscus* Cao & Li, **sp. n.** (♂). Females of *Bavia capistrata* (C.L. Koch, 1846) and *Phintella suavisoides* Lei & Peng, 2013 are described for the first time. DNA barcodes of 12 species were obtained for future use.

## Introduction

Of 598 salticid genera and 5912 species known worldwide (World Spider Catalog 2016), 95 genera and 473 species are recorded from China, with 44 genera and 94 species recorded from Yunnan (Li and Lin 2016). The lists are far from being complete as large parts of the country are still poorly studied.

Being a border area with Vietnam, Laos and Myanmar, Yunnan shares the jumping spider taxa with those countries, of which the fauna of Vietnam is the best studied, with 56 genera and 116 species (Ono et al. 2012), the majority described and recorded by Żabka (1985). From other countries, 18 species are known from Laos and 55 from Myanmar (World Spider Catalog 2016).

While studying spiders in Xishuangbanna in South Yunnan, 21 salticid species were found. The goal of this paper is to report these species, including descriptions of 19 new species and the redescriptions of two known species.

## Material and methods

The material came from Xishuangbanna in South Yunnan (21°08'N–22°36'N, 99°56'E–101°50'E). The area belongs to the transitional zone from tropical South to subtropical East Asia (Zhu et al. 2004). The region has an area of 19,120 km^2^, with mountain ridges running north-south, and the elevation decreasing southwards. The current study is based on 10 years of collecting in Xishuangbanna. More details on the spider diversity in the area and collection methods can be found in Zheng et al. (2015).

The specimens were preserved in 95% ethanol and were examined and measured with Olympus SZX12 and BX41 microscopes. Photos were taken with an Olympus C7070 wide zoom digital camera mounted on an Olympus SZX12 stereomicroscope. The images were processed with Helicon image stacking software. Vulvae were removed and digested with lactic acid or a 10% warm solution of potassium hydroxide (KOH). All measurements are in millimetres. References to figures in the cited papers are listed in lowercase type (fig. or figs); figures in this paper are noted with an initial capital (Fig. or Figs).

### Abbreviations used

AER
 anterior eye row 


ALE
 anterior lateral eyes 


AL
 abdomen length 


AME
 anterior median eyes 


AW
 abdomen width 


CD
 copulatory ducts 


CL
 carapace length 


CO
 copulatory opening 


CW
 carapace width 


DB
 dorsal-basal bulge 


DTA
 dorsal tibial apophysis 


E
 embolus 


EB
 embolus base 


EC
 extension of cymbium 


EFL
 length of eye field 


FD
 fertilization ducts 


H
 hood 


LP
 lamellar process 


P
 pocket 


PE
 posterior extension 


PER
 posterior eye row 


PLE
 posterior lateral eyes 


PME
 posterior median eyes 


PP
 prolateral process 


R
 receptacles 


RBB
 retrolateral bulbal bump 


RP
 retrolateral process 


RTA
 retrolateral tibial apophysis 


RVA
 retrolateral ventral tibial apophysis 


S
 serration 


SA
 sclerotized apophysis 


SD
 seminal duct 


RP
 retrolateral process 


SDA
 seminal duct angle 


TA
 tegular apophysis 


TD
 translucent duct 


TP
 tegulum protrusion 


VTA
 ventral tibial apophysis 


The leg spination pattern is given after Platnick and Shadab (1975): d, p, v, r for dorsal, prolateral, ventral and retrolateral sides of a segment.

For 12 species the DNA barcodes were obtained for future use (the samples collected in 2006, 2007 and 2009 were not extracted successfully). A partial fragment of the mitochondrial gene cytochrome oxidase subunit I (COI) was amplified and sequenced following the protocol in Miller et al. (2010). Primers used in this study are: LCO1490 (5’-CWACAAAYCATARRGATATTGG-3’) and HCO-N-2198 (5’- TAAACTTCAGGGTGACCAAAAAATCA-3’) (Folmer et al. 1994). Voucher information and GenBank accession number for all samples are listed in Table 1. All specimens, including voucher specimens, are deposited in the Institute of Zoology, Chinese Academy of Sciences (IZCAS) in Beijing, China.

**Table 1.** Voucher specimen information,

Species	Sequence length	GenBank accession number	Collecting localities in Xishuangbanna
*Agorius tortilis* sp. n.	629 bp	KU893260	Mengyang, Jinghong
*Bavia capistrata*	629 bp	KU893261	Menglun, Mengla
*Carrhotus kevinlii* sp. n.	629 bp	KU893263	Menglun, Mengla
*Carrhotus sarahcrewsae* sp. n.	629 bp	KU893264	Menglun, Mengla
*Chinattus wengnanensis* sp. n.	629 bp	KU893265	Menga, Jinghong
*Chinophrys mengyangensis* sp. n.	629 bp	KU893266	Mengyang, Jinghong
*Cocalus menglaensis* sp. n.	629 bp	KU893267	Xiaolongha, Mengla
*Cosmophasis xiaolonghaensis* sp. n.	629 bp	KU893268	Xiaolongha, Mengla
*Nannenus menghaiensis* sp. n.	629 bp	KU893269	Menghai, Jinghong
*Phintella lepidus* sp. n.	629 bp	KU893270	Mengyang, Jinghong
*Phintella suavisoides*	629 bp	KU893271	Menglun, Mengla
*Ptocasius paraweyersi* sp. n.	629 bp	KU893272	Menglun, Mengla

## Taxonomy

### Family Salticidae Blackwall, 1841

#### Genus *Afraflacilla* Berland & Millot, 1941

##### Afraflacilla ballarini

Taxon classificationAnimaliaAraneaeSalticidae

Cao & Li sp. n.

http://zoobank.org/898EE80F-6D26-4FFB-B9F4-832A81944515

Figs 1 , 2 , 43

###### Type.

**Holotype** ♂: CHINA, Yunnan, Mengla County, Menglun Town, rubber plantation (21°54.684'N, 101°16.319'E, 585 m), 7 March 2006, G. Zheng leg.

###### Etymology.

The new species is named after Francesco Ballarin (IZCAS) for his study on the spiders of Asia; noun (name) in genitive case.

###### Diagnosis.

**Figure 1.**
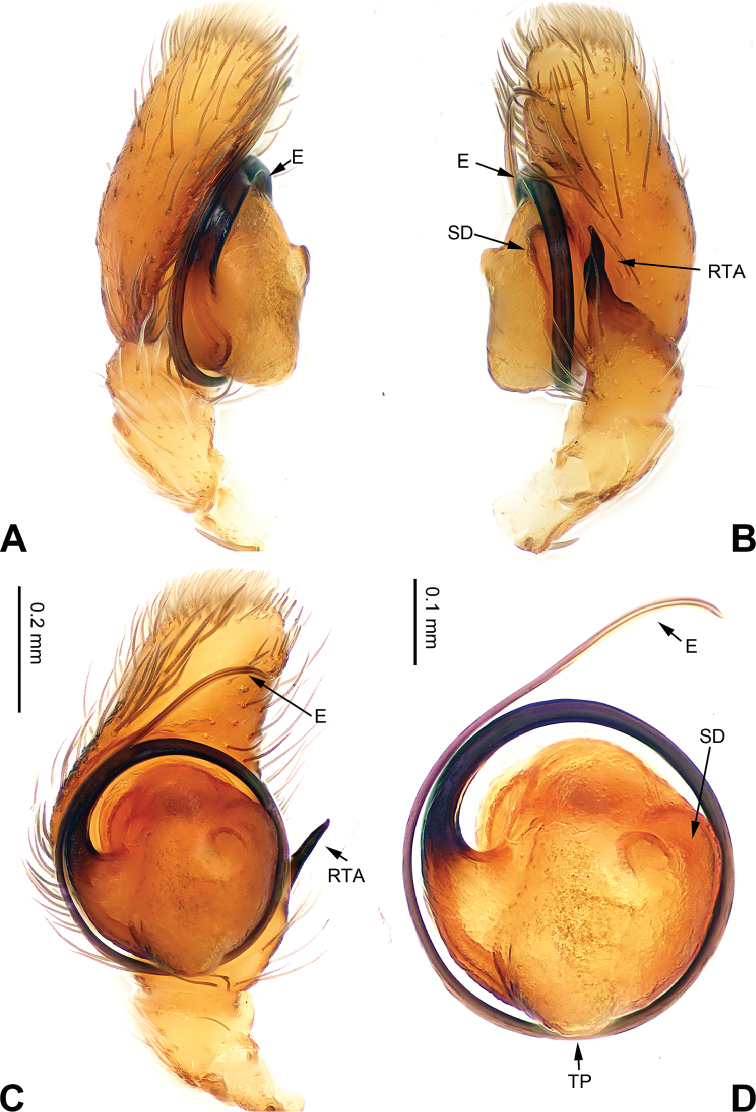
Palp of *Afraflacilla ballarini* sp. n., male holotype. **A** prolateral **B** retrolateral **C** ventral **D** bulb, ventral. Scale bar equal for **A–C**.

**Figure 2.**
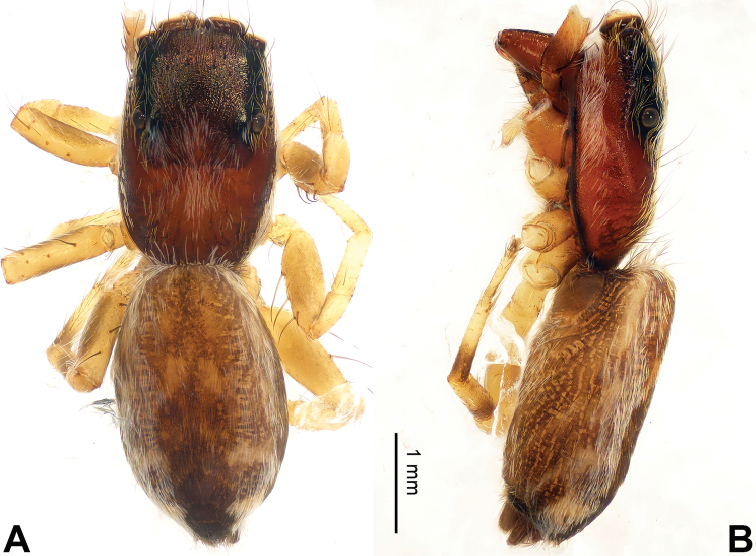
Habitus of *Afraflacilla ballarini* sp. n., male holotype. **A** dorsal **B** lateral.

The male resembles *Afraflacilla grayorum* Żabka, 1993 (Żabka 1993: figs 7A–D, 8A–C) by having similar embolus (Fig. 1C–D) and body shape (Fig. 2), but differs in the shape of the tegulum (Fig. 1D) and embolus set at nine o’clock vs. six o’clock in *Afraflacilla grayorum*. Also, the course of the seminal duct and tegular protrusion are in different positions (six o’clock vs. four o’clock in *Afraflacilla grayorum*). The tibial apophysis lacks a dorsolateral protrusion (vs. this character in *Afraflacilla grayorum*) (Fig. 1B).

###### Description.

**Male** (holotype). Total length 3.85, CL 1.85, CW 1.20, AL 2.00, AW 1.40. Eye measurements: AME 0.32 ALE 0.18 PME 0.08 PLE 0.15; AER 1.00, PER 1.00, EFL 0.85. Clypeus 0.12 high. Legs: I 3.58 (1.05, 0.70, 0.85, 0.60, 0.38); II 2.31 (0.78, 0.33, 0.50, 0.40, 0.30); III 2.60 (0.80, 0.35, 0.55, 0.50, 0.40); IV 3.34 (1.00, 0.50, 0.75, 0.64, 0.45).

Carapace brown with grey and white hairs (Fig. 2A). Sides and clypeus with white marginal band. Ocular area dark brown. Chelicerae and labium brown. *Maxillae* brown with white tips. Sternum greyish brown. Abdomen oval, brownish, anterior and sides with white hairs. Venter and spinnerets dark brown. Leg I more robust and darker than the other legs, which are yellowish. Spination of leg I: femur d2-1-1; tibia p0-1-0, metatarsus v2-0-2. Palpal tibia short, about 1/3 length of cymbium. Cymbial tip about 1.5 times as long as tibia. RTA pointed, subequal to the length of the tibia (Fig. 1B). Bulb oval, with blunt outgrowth and posterior protrusion (Fig. 1D). Seminal duct with loops. Embolus elongate, starting at nine o’clock and coiled more than once around the bulb (Fig. 1C).

**Female.** Unknown.

###### Distribution.

Known only from the type locality.

#### Genus *Agorius* Thorell, 1877

##### Agorius tortilis

Taxon classificationAnimaliaAraneaeSalticidae

Cao & Li sp. n.

http://zoobank.org/4BB85C25-617E-4601-9310-F134823897BE

Figs 3 , 4 , 43

###### Type.

**Holotype** ♂: CHINA, Yunnan, Jinghong City, Mengyang Town, tunnel in Mt. Baihuashan (22°69.529'N, 101°55.210'E, 856 m), 16 July 2012, Q. Zhao & Z. Chen leg. **Paratype**: 1♀, same data as holotype.

###### Etymology.

From Latin *tortilis* (coiled), in reference to the shape of embolus; adjective.

###### Diagnosis.

**Figure 3.**
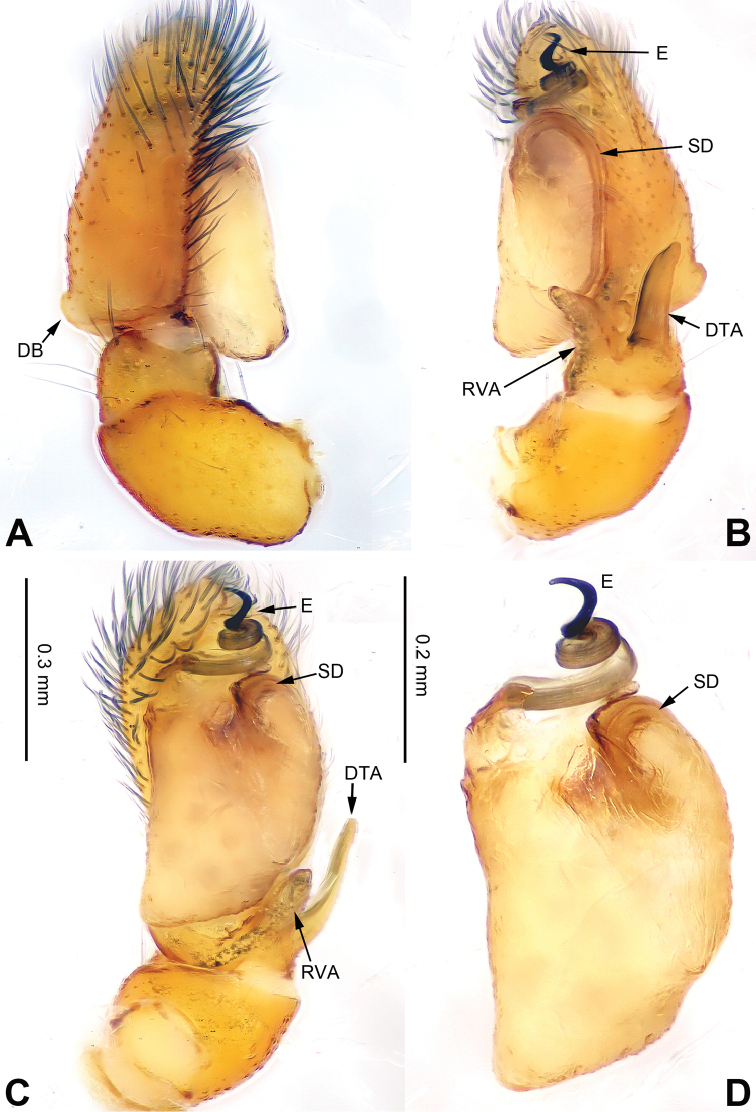
Palp of *Agorius tortilis* sp. n., male holotype. **A** prolateral **B** retrolateral **C** ventral **D** bulb, ventral. Scale bar equal for **A–C**.

**Figure 4.**
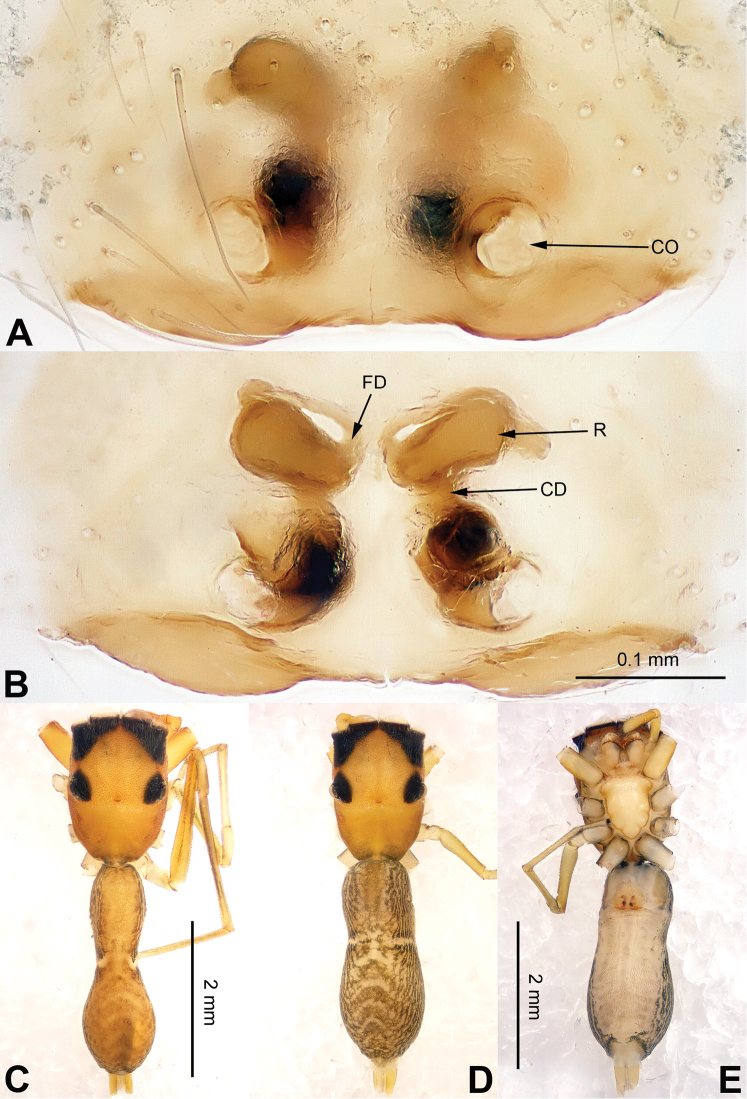
*Agorius tortilis* sp. n., female paratype and male holotype. **A** epigyne, ventral **B** vulva, dorsal **C** male habitus, dorsal **D** female habitus, dorsal **E** female habitus, ventral. Scale bar equal for **A** and **B**; equal for **D** and **E**.

The male is similar to that of *Agorius lindu* Prószyński, 2009 (Prószyński 2009: figs 7–8, 29–30, 54, 59) by body shape (Fig. 4C) and tegulum (Fig. 3C–D), but the embolus has 3 coils (Fig. 3D) vs. 1; dorsal-retrolateral tibial apophysis lacking, without terminal hook (Fig. 3B), which is present in *Agorius lindu*. The female differs from that of *Agorius lindu* by the shape of the copulatory openings (Fig. 4A), which are small circular holes vs. slanted ovals in *Agorius lindu*, and the copulatory openings are separated by about two diameters vs. only 1/4 diameter in *Agorius lindu*.

###### Description.

**Male** (holotype). Total length 5.23, CL 1.83, CW 1.50, AL 3.40, AW 1.09. Eye measurements: AME 0.48, ALE 0.27, PME 0.01, PLE 0.25, AER 1.48, PER 1.49, EFL 1.47. Clypeus 0.04 high. Legs: I 18.26 (3.28, 4.00, 2.64, 0.50, 0.46); II 3.95 (1.55, 0.55, 1.31, 1.06, 0.45); III missing; IV 7.50 (2.13, 0.73, 2.13, 1.88, 0.63).

Carapace greyish-yellow (Fig. 4C). *Chelicerae* yellow, sparsely covered with fine grey hairs. *Maxillae* yellow with black anterior margin and grey hairs on inner margins. *Labium* dark yellow, tip with black hairs. *Sternum* yellowish. Abdomen thin, elongate with median constriction. Venter and spinnerets yellow. Legs I thin and long, especially the patella. Spination of leg I: tibia v2-2-2-2; metatarsus p2-0-2. Palp: patella large, thicker than tibia. Tibia with two large apophyses, RVA and DTA (Fig. 3B). Cymbium with dorsal-basal small bulge (Fig. 3A). Tegulum with a broad prolateral flap. Seminal duct encircling retrolateral part of tegulum. Embolus with tapering spiral (Fig. 3C).

**Female** (paratype). Total length 5.54, CL 2.35, CW 1.38, AL 3.19, AW 1.23. Eye measurements: AME 0.44, ALE 0.25, PME 0.01, PLE 0.25, AER 1.30, PER 1.32, EFL 1.36. Clypeus 0.04 high. Legs: I 7.64 (2.50, 2.56, 1.80, 0.40, 0.38); II 4.15 (1.25, 0.56, 1.04, 0.90, 0.40); III missing; IV 4.31 (1.22, 0.56, 1.09, 1.00, 0.44).

Abdomen higher and broader than in male, other characters similar. Epigyne heavily sclerotised along the posterior margin (Fig. 4A). Copulatory opening grooves round and separated from each other by two diameters, located 1 diameter from the posterior margin. Vulva: copulatory ducts short and sclerotised, anterior part thicker than the posterior. Receptacles pyriform. Fertilisation ducts elongate and located at the posterior part of the receptacles (Fig. 4B).

###### Distribution.

Known only from the type locality.

#### Genus *Bavia* Simon, 1877

##### Bavia capistrata

Taxon classificationAnimaliaAraneaeSalticidae

(C.L. Koch, 1846)

Figs 5 , 6 , 43

Maevia capistrata C.L. Koch, 1846: 76, fig. 1331 (♂).
Bavia capistrata : Żabka 1988: 435, figs 37–39 (♂, removed from synonymy with Evarcha flavocincta).

###### Material examined.

1♂, CHINA, Yunnan, Mengla County, Menglun Town, Xishuangbanna Nature Reserve: nearby fish pond (21°57.883'N, 101°12.147'E, 839 m), ravine rainforest, 15 August 2011, Q. Zhao & Z. Chen leg.; 1♀, CHINA, Yunnan, Mengla County, Xiaolongha Village, Xishuangbanna Nature Reserve: Biological diversity corridor (21°24.265'N, 101°37.300'E, 653 m), seasonal rainforest, 27 June 2012, Q. Zhao & Z. Chen leg.

###### Diagnosis.

**Figure 5.**
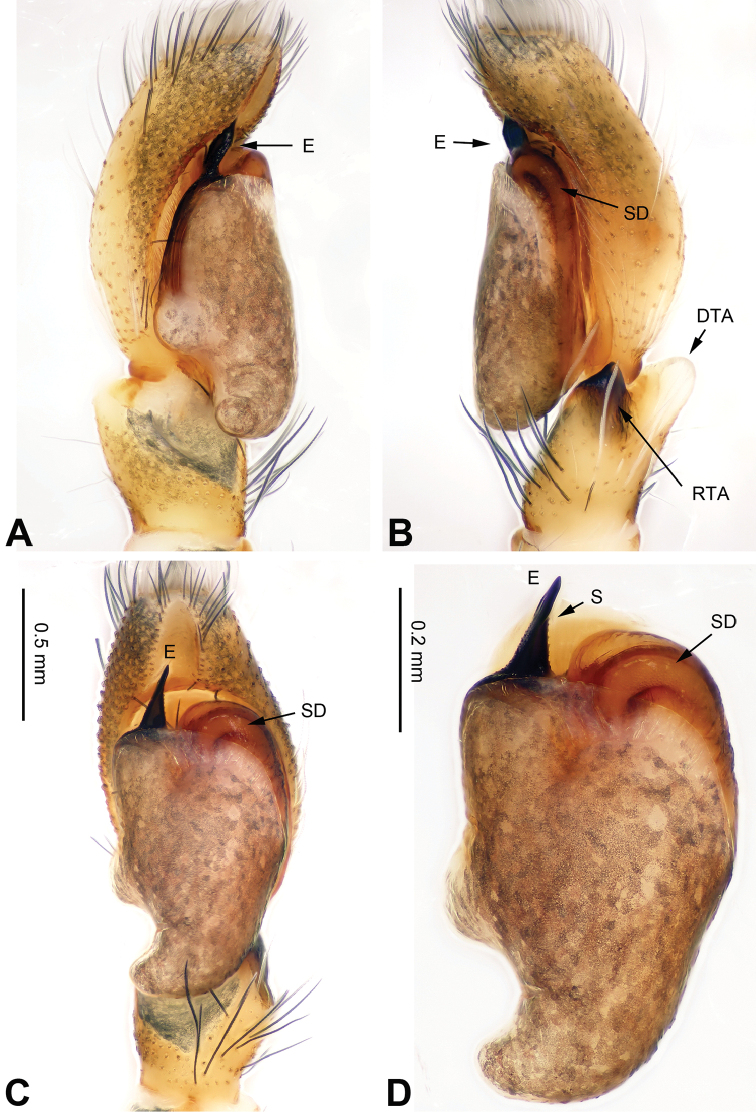
Palp of *Bavia capistrata*, male from Xishuangbanna. **A** prolateral **B** retrolateral **C** ventral **D** bulb, ventral. Scale bar equal for **A–C**.

**Figure 6.**
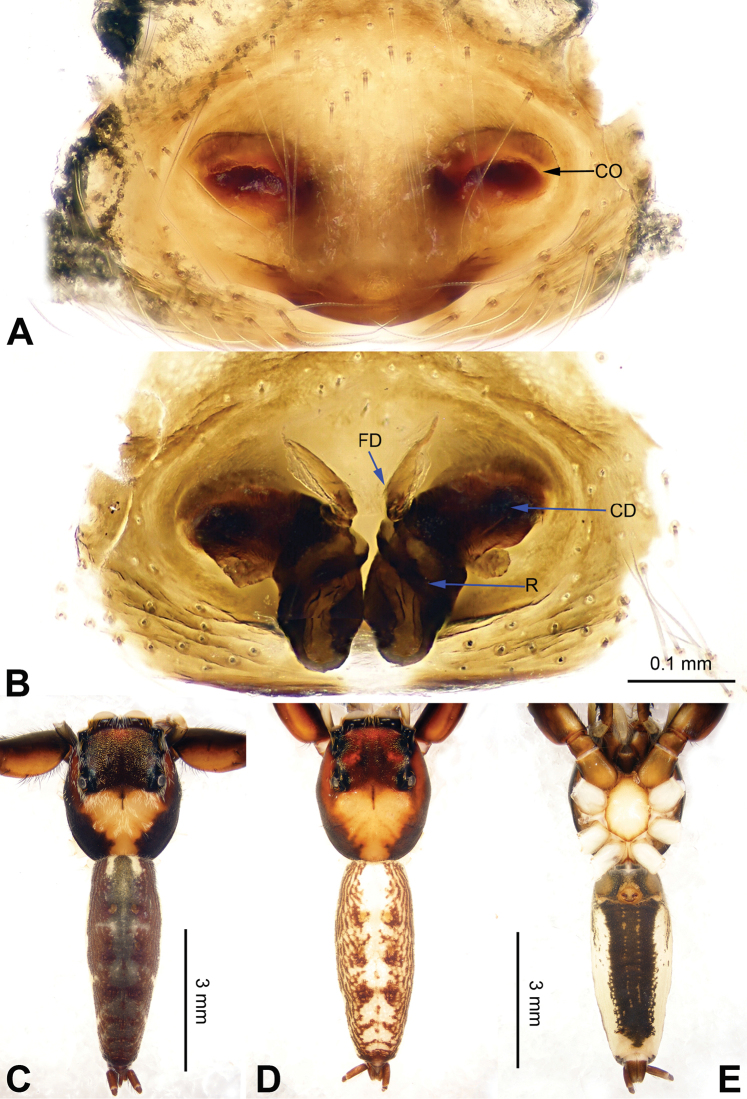
*Bavia capistrata*, female and male from Xishuangbanna. **A** epigyne, ventral **B** vulva, dorsal **C** male habitus, dorsal **D** female habitus, dorsal **E** female habitus, ventral. Scale bars equal for **A** and **B**; equal for **D** and **E**.

Differs from the closely related *Bavia aericeps* Simon, 1877 (see Żabka 1988: figs 29–36) by the tibia with a distinct dorsal apophysis (Fig. 5B) and serrated embolus (Fig. 5D). The females differ from *Bavia aericeps* by the horizontal position of the copulatory openings (Fig. 6A) vs. the inclined copulatory organs and the arc-shaped posterior epigynal margin (Fig. 6A) vs. triangular.

###### Description.

**Male.** Well described by Żabka (1988).

**Female.** Total length 8.70, CL 3.44, CW 2.75, AL 5.26, AW 1.92. Eye measurements: AME 0.69, ALE 0.28, PME 0.06, PLE 0.29, AER 2.00, PER 2.00, EFL 1.88. Clypeus 0.16 high. Legs: I 6.92 (2.13, 1.35, 1.75, 1.14, 0.55); II 5.50 (1.70, 1.06, 1.26, 0.98, 0.50); III 5.33 (1.60, 0.95, 1.00, 1.19, 0.59); IV 9.09 (2.25, 1.00, 1.74, 1.74, 0.63).

Carapace reddish-brown, lighter dorsally, ocular area dark brown (Fig. 6C) with white setae. Chelicerae dark brown. *Maxillae* elongate with white tips. Labium dark brown with white tips. Sternum yellowish. Abdomen long with light broad median stripe and grey margins. Venter with few longitudinal rows of white dots. Spinnerets brownish grey. Legs I more robust and darker than others. Legs II–IV yellowish. Spination of leg I: femur d2-1-0; tibia v2-2-2, metatarsus p2-0-2. Epigyne strongly sclerotised along the posterior midline margin (Fig. 6A). Copulatory openings slit shaped, with strongly sclerotised edges. Distance between the openings subequal to 1.5 times the length of a copulatory opening. Copulatory ducts short and strongly sclerotised, receptacles close to each other. The length and width of receptacles subequal to the copulatory ducts. Fertilisation ducts located at the joined part of the copulatory ducts and receptacles (Fig. 6B).

###### Distribution.

Malaysia to Australia, Pacific Islands, and South China.

###### Remark.

Female of *Bavia capistrata* is described for the first time.

##### Bavia exilis

Taxon classificationAnimaliaAraneaeSalticidae

Cao & Li sp. n.

http://zoobank.org/2DC9C8CF-27F1-4E1C-86D4-F4303CE1C907

Figs 7 , 8 , 43

###### Type.

**Holotype** ♂: CHINA, Yunnan, Mengla County, Xiaolongha Village, Xishuangbanna Nature Reserve: Biological diversity corridor (21°24.230'N, 101°36.262'E, 715 m), seasonal rainforest, 4 June 2012, Q. Zhao & Z. Chen leg.

###### Etymology.

From Latin *exilis* (slender), in reference to the shape of the abdomen; adjective.

###### Diagnosis.

**Figure 7.**
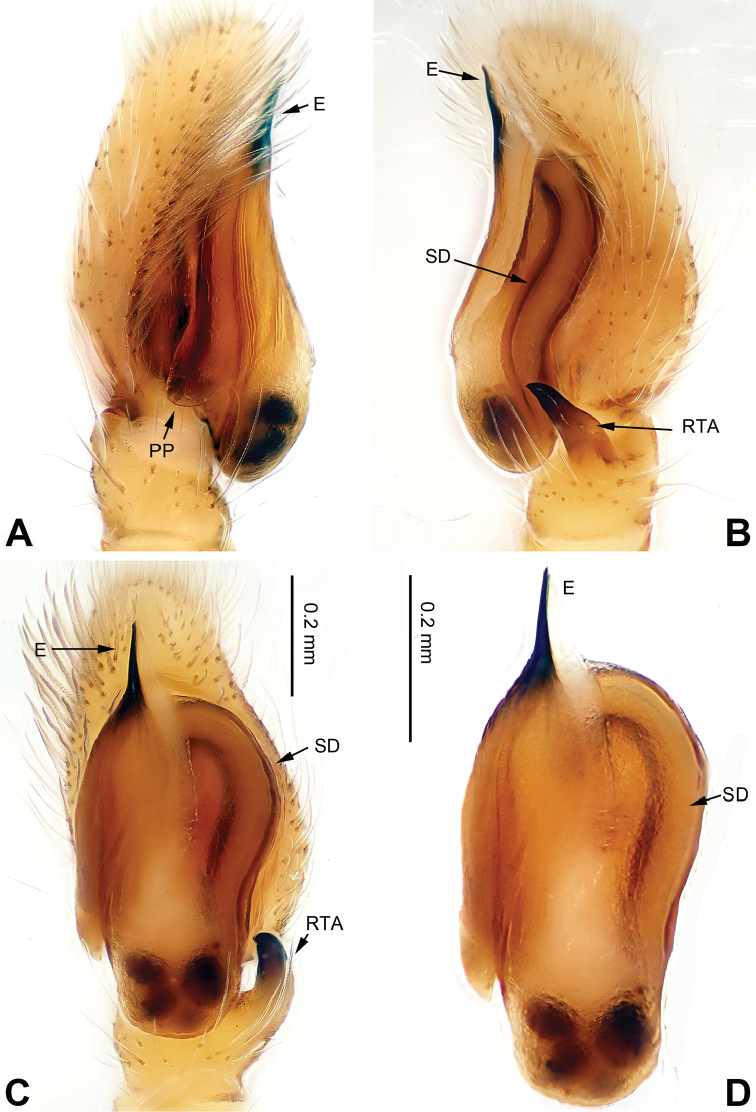
Palp of *Bavia exilis* sp. n., male holotype. **A** prolateral **B** retrolateral **C** ventral **D** bulb, ventral. Scale bar equal for **A–C**.

Similar to *Bavia aericeps*, but RTA much larger (Fig. 7B) and embolus with retrolateral membranous margin (Fig. 7C). Compared to *Bavia capistrata* (Fig. 5B–D), the tibia lacks a distinct dorsal apophysis, and the embolus is not serrated.

###### Description.

**Male** (holotype). Total length 6.65, CL 2.75, CW 1.75, AL 3.90, AW 1.10. Eye measurements: AME 0.60, ALE 0.21, PME 0.06, PLE 0.16, AER 1.52, PER 1.55, EFL 1.41. Clypeus 0.08 high. Legs: I 6.15 (1.75, 1.00, 1.55, 1.00, 0.85); II 4.00 (1.15, 0.70, 0.85, 0.70, 0.60); III 3.55 (1.00, 0.60, 0.65, 0.80, 0.50); IV 4.35 (1.25, 0.65, 0.90, 1.00, 0.55).

**Figure 8.**
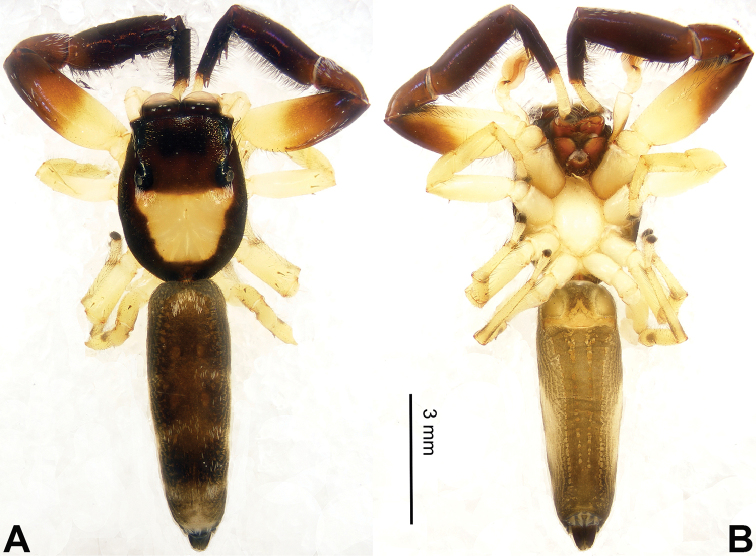
Habitus of *Bavia exilis* sp. n., male holotype. **A** dorsal **B** ventral.

Carapace dark brown with central lighter trapezoid dorsally (Fig. 8A). Chelicerae brown. Maxillae and labium brown, light tips with greyish-brown hairs. Sternum yellowish. Abdomen elongate, slender and grey. Venter and spinnerets dark greyish. Legs I more robust and darker than others, legs II–IV yellowish. Spination of leg I: femur d1-0-0; tibia v2-2-2; metatarsus v2-0-2. Palp: tibia short, about 1/4 length of cymbium. Tibial apophysis bent, subequal to the length of tibia (Fig. 7B). Embolus short and pointed, with retrolateral membranous margin (Fig. 7C).

**Female.** Unknown.

###### Distribution.

Known only from the type locality.

#### Genus *Carrhotus* Thorell, 1891

##### Carrhotus kevinlii

Taxon classificationAnimaliaAraneaeSalticidae

Cao & Li sp. n.

http://zoobank.org/C2408FE6-35E7-45B4-9858-FE61B1747C3B

Figs 9 , 10 , 43

###### Type.

**Holotype** ♂: CHINA, Yunnan, Mengla County, Menglun Town, Lüshilin (21°54.398'N, 101°16.754'E, 705 m), seasonal rainforest, 19 August 2011, K. Li leg. **Paratypes**: 1♂, same data as holotype; 1♂2♀, CHINA, Yunnan, Mengla County, Xiaolongha Village, Xishuangbanna Nature Reserve: Biological diversity corridor (21°24.213'N, 101°36.995'E, 834 m), seasonal rainforest, 3 June 2012, Q. Zhao & Z. Chen leg.

###### Etymology.

The new species is named after Mr Kevin Li (=Kaiwen Li) for his assistance in field work; noun (name) in genitive case.

###### Diagnosis.

**Figure 9.**
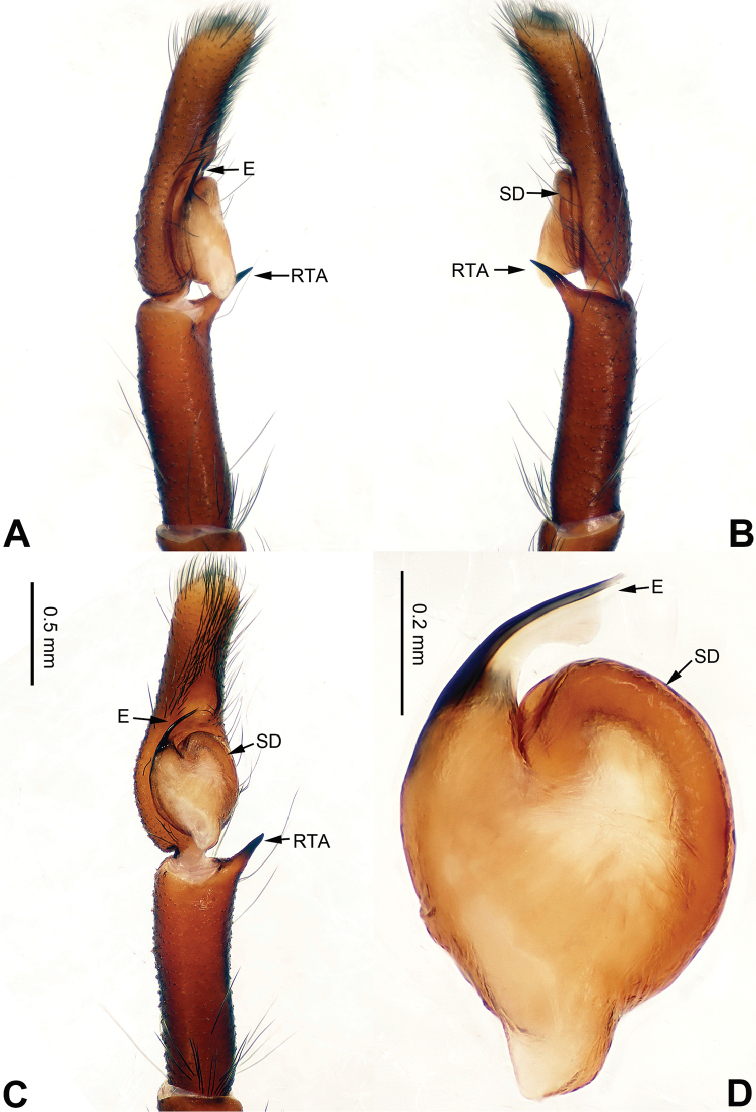
Palp of *Carrhotus kevinlii* sp. n., male holotype. **A** prolateral **B** retrolateral **C** ventral **D** bulb, ventral. Scale bar equal for **A–C**.

**Figure 10.**
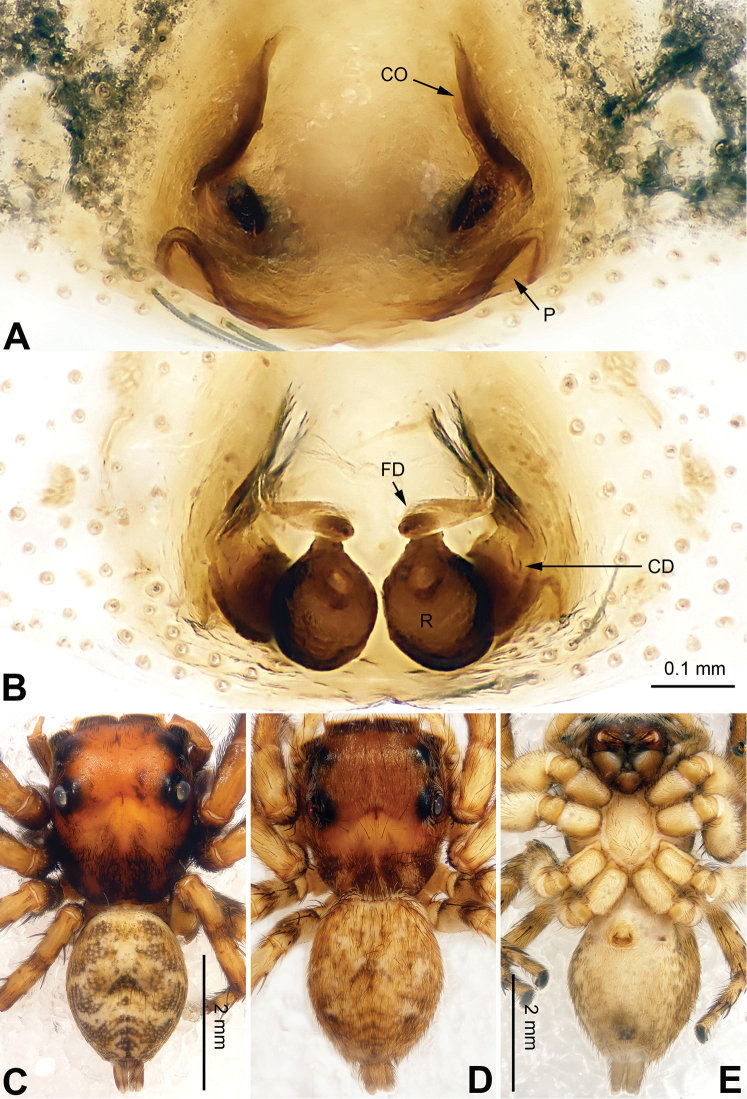
*Carrhotus kevinlii* sp. n., female paratype and male holotype. **A** epigyne, ventral **B** vulva, dorsal **C** male habitus, dorsal **D** female habitus, dorsal **E** female habitus, ventral. Scale bars equal for **A** and **B**; equal for **D** and **E**

Cymbium twice as long as bulb (Fig. 9C) vs. approximately equal to bulb length in other congeners and embolus accompanied by a membrane (Fig. 9D). The female resembles *Carrhotus viduus* (see Prószyński 2009b: figs 22–23), but the copulatory openings are highly sclerotised (Fig. 10A) vs. oval in *Carrhotus viduus*. Also, the receptacles are subglobose (Fig. 10B) vs. irregular in *Carrhotus viduus*.

###### Description.

**Male** (holotype). Total length 6.08, CL 3.20, CW 2.64, AL 2.88, AW 1.95. Eye measurements: AME 0.55, ALE 0.24, PME 0.08, PLE 0.37, AER 2.41, PER 2.35, EFL 2.00. Clypeus 0.24 high. Legs: I 5.93 (1.85, 1.10, 1.38, 0.82, 0.78); II 5.86 (1.80, 1.12, 1.30, 0.86, 0.78); III 6.37 (2.15, 1.00, 1.36, 1.26, 0.60); IV 6.48 (0.70, 1.47, 1.41, 0.94, 1.96).

Carapace brown (Fig. 10C). Lower margin and area around eyes darker with white hairs, clypeus brown. Chelicerae dark brown, *maxillae* and labium brown with white tips. Sternum yellowish. Abdomen grey with irregular beige patches and beige sides, entire surface covered with a few short, whitish hairs. Venter grey. Spinnerets grey-brown. Legs dark brown. Spination of leg I: femur d3-1-1; patella p0-1-0, r0-1-0; tibia v2-2-2, p2-0-1, r1-0-0; metatarsus v2-0-2, p1-0-0. Palpal tibia and cymbium long, tibia about 4/5 length of cymbium. Retrolateral apophysis almost as wide as tibia, with pointed tip (Fig. 9B). Cymbium with long and dark brown bristles. Bulb about half the length of the cymbium. Embolus with membrane, bow-shaped, and subequal to half the length of the tegulum (Fig. 9C).

**Female** (one of paratypes) very similar to the male, with clypeus brown and legs light brown. Total length 7.05, CL 3.25, CW 2.66, AL 3.80, AW 2.97. Eye measurements: AME 0.75, ALE 0.39, PME 0.08, PLE 0.37, AER 2.45, PER 2.55, EFL 1.36. Clypeus 0.24 high. Legs: I 6.17 (1.90, 1.28, 1.41, 0.95, 0.63); II 5.68 (1.84, 1.22, 1.20, 0.79, 0.63); III 6.66 (2.15, 1.02, 1.36, 1.33, 0.80); IV 7.07 (2.15, 1.22, 1.41, 1.45, 0.84). Spination of leg I: femur d3-1-1; patella p0-1-0, r0-1-0; tibia v2-2-2, p1-0-0, r1-0-0; metatarsus v2-0-2. Copulatory openings slit shaped with strongly sclerotised edges. The distance between openings subequal to the length of openings (Fig. 10A). Copulatory ducts short and broad. Receptacles subglobular and diameter equal to the width of copulatory ducts. Fertilisation ducts located at the anterior part of the receptacles (Fig. 10B).

###### Distribution.

Known from several localities in Xishuangbanna.

##### Carrhotus sarahcrewsae

Taxon classificationAnimaliaAraneaeSalticidae

Cao & Li sp. n.

http://zoobank.org/E1FE66AD-EFBB-4913-9F55-C7708AC8A80C

Figs 11 , 12 , 43

###### Type.

**Holotype** ♂: CHINA, Yunnan, Mengla County, Menglun Town, 48 km landmark in Nature Reserve (21°38.853'N, 101°09.625'E, 1001 m), seasonal rainforest, 30 July 2012, Q. Zhao & Z. Chen leg.

###### Etymology.

The new species is named after Dr Sarah Crews for her contribution to the study of the spider family Selenopidae; noun (name) in genitive case.

###### Diagnosis.

**Figure 11.**
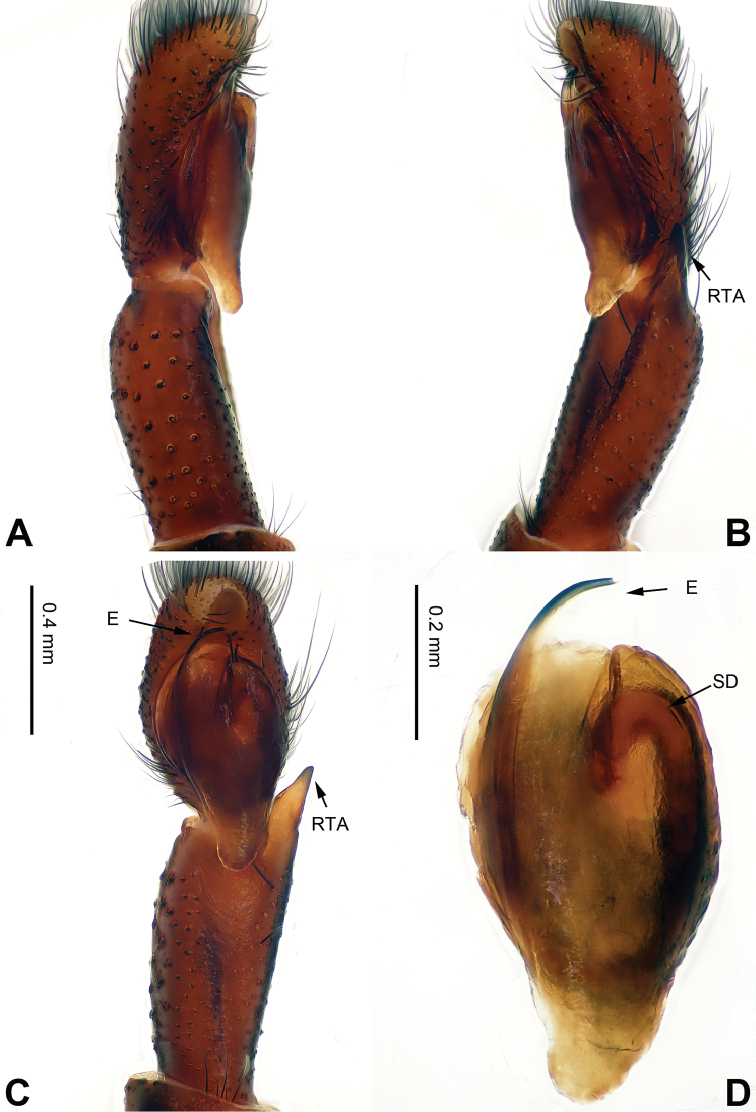
Palp of *Carrhotus sarahcrewsae* sp. n., male holotype. **A** prolateral **B** retrolateral **C** ventral **D** bulb, ventral. Scale bar equal for **A–C**.

The male resembles that of *Carrhotus sannio* (see Żabka 1985: figs 63–65), but the length of the tibia is nearly equal to the cymbium (Fig. 11A) vs. 2/3 of the length of the cymbium in *Carrhotus sannio*, and the cymbium has no apical process (Fig. 11C). Compared to *Carrhotus kevinlii* sp. n., the bulb length is subequal to the cymbium vs. less than half the length, the embolus has no membrane and the angle between the RTA and tibia is early 20° vs. about 45° in *Carrhotus kevinlii* sp. n.

###### Description.

**Male** (holotype).Total length 5.77, CL 2.97, CW 2.25, AL 2.80, AW 1.94. Eye measurements: AME 0.63, ALE 0.29, PME 0.05, PLE 0.25, AER 1.84, PER 1.70, EFL 1.64. Clypeus 0.25 high. Legs: I 8.22 (2.25, 1.40, 2.13, 1.64, 0.80); II 6.38 (1.96, 1.06, 1.48, 1.25, 0.63); III 6.44 (2.00, 1.00, 1.33, 1.30, 0.81); IV 6.47 (1.93, 1.00, 1.41, 1.34, 0.79).

**Figure 12.**
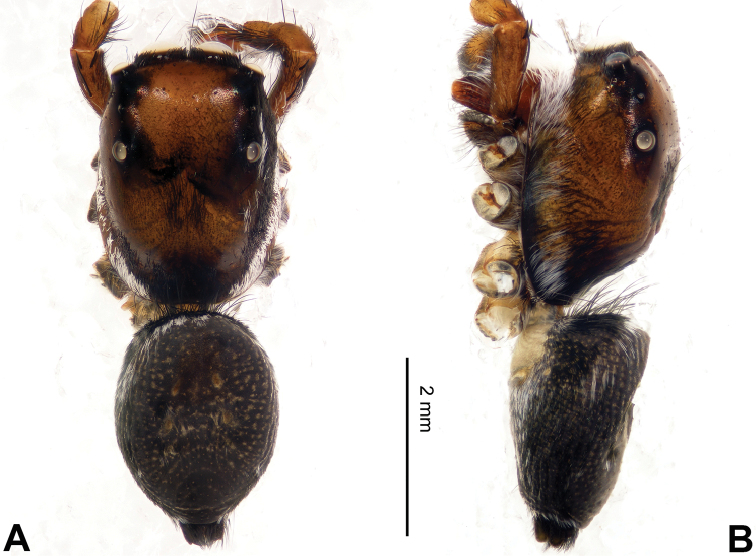
Habitus of *Carrhotus sarahcrewsae* sp. n., male holotype. **A** dorsal **B** lateral. Scale bar equal for **A** and **B**.

Carapace dark brown, margin and area around eyes dark with white hairs (Fig. 12A). Chelicerae dark brown, with dense greyish hairs. *Maxillae* and labium dark brown, tips with dark setae. Sternum light brown. Abdomen dark grey with irregular beige patches. Venter and spinnerets dark greyish. Legs brown. Spination of leg I: femur d3-1-1; patella p0-1-0; tibia v2-2-2, p2-0-2, r2-0-2; metatarsus v2-0-2, p1-0-1, r1-0-1. Palp: tibia subequal to the length of cymbium. Cymbium with long, dark brown bristles. Tibial apophysis triangular (Fig. 11B). Bulb equal to the length of the cymbium. Embolus short, about 1/4 the length of the tegulum, bent (Fig. 11D).

**Female.** Unknown.

###### Distribution.

Known only from the type locality.

#### Genus *Chinattus* Logunov, 1999

##### Chinattus wengnanensis

Taxon classificationAnimaliaAraneaeSalticidae

Cao & Li sp. n.

http://zoobank.org/1E75B981-6EB3-4955-9223-126169A81614

Figs 13 , 14 , 43

###### Type.

**Holotype** ♂: CHINA, Yunnan, Jinghong, Menga Town, Wengnan Village (22°05.020'N, 100°22.087'E, 1118 m), secondary forest, 24 July 2012, Q. Zhao & Z. Chen leg. **Paratypes**: 2♀, same data as holotype; 1♂, CHINA, Yunnan, Mengla County, Menglun Town, 48 km landmark in Nature Reserve (21°53.997'N, 101°16.957'E, 593 m), secondary forest, 11 August 2011, Q. Zhao & Z. Chen leg.

###### Etymology.

The species name is derived from the name of type locality; adjective.

###### Diagnosis.

**Figure 13.**
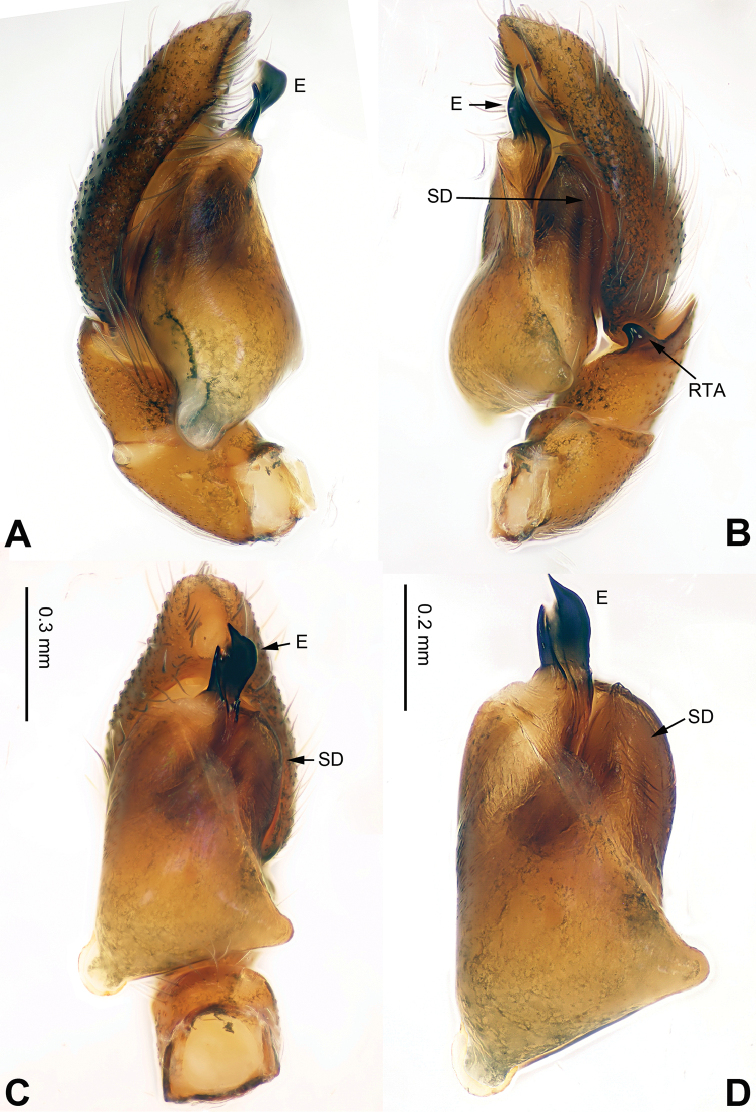
Palp of *Chinattus wengnanensis* sp. n., male holotype. **A** prolateral **B** retrolateral **C** ventral **D** bulb, ventral. Scale bar equal for **A–C**.

**Figure 14.**
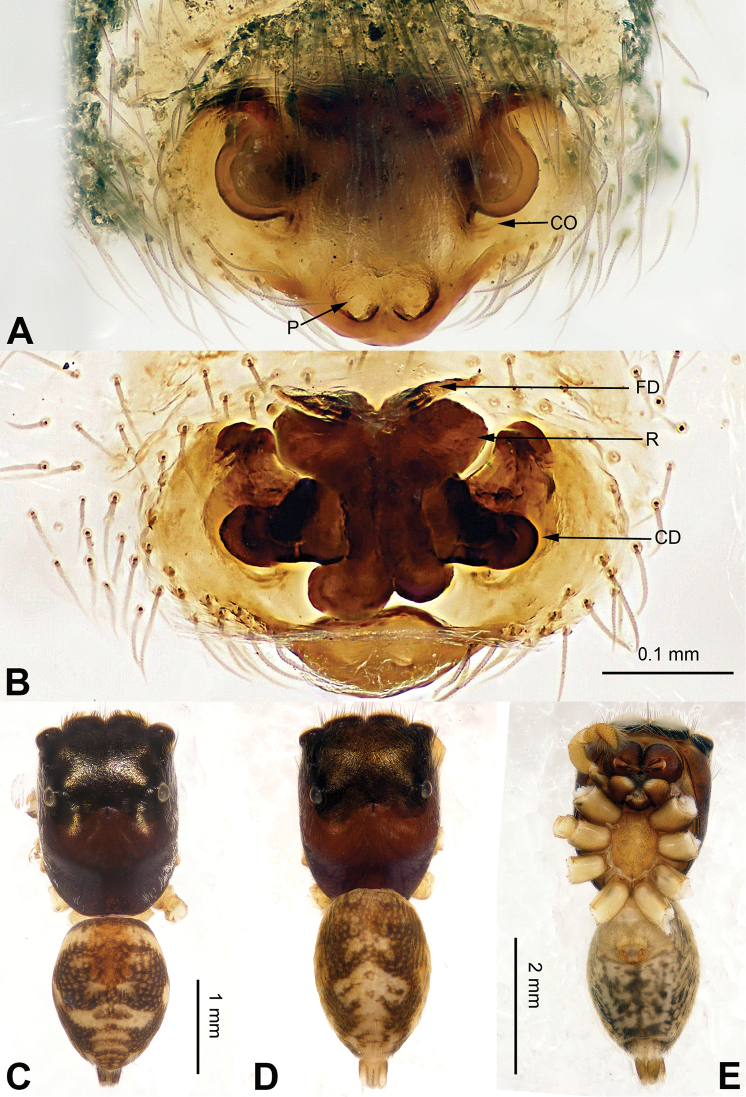
*Chinattus wengnanensis* sp. n., female paratype and male holotype. **A** epigyne, ventral **B** vulva, dorsal **C** male habitus, dorsal **D** female habitus, dorsal **E** female habitus, ventral. Scale bars equal for **A** and **B**; equal for **D** and **E**.

The male can be distinguished from the other congeners by the broad and bifurcate embolus and the nearly rectangular tegulum (in ventral view) (Fig. 13C–D). The female is similar to *Carrhotus undulatus* (Song & Chai, 1992) (see Prószyński 1992: figs 22–27), but the copulatory openings have a semicircular highly-sclerotised lobe (Fig. 14A), epigyne with posterior projection and two pockets vs. only one in *Carrhotus undulatus* (Fig. 14A).

###### Description.

**Male** (holotype). Total length 4.25, CL 2.25, CW 1.75, AL 2.00, AW 1.44. Eye measurements: AME 0.49, ALE 0.34, PME 0.09, PLE 0.24, AER 1.72, PER 1.56, EFL 1.40. Clypeus 0.13 high. Legs: I 4.85 (1.50, 0.86, 1.13, 0.80, 0.56); II 5.93 (1.25, 0.69, 0.85, 0.70, 0.44); III 4.73 (1.56, 0.80, 0.90, 1.00, 0.47); IV 4.40 (1.41, 0.59, 0.90, 1.00, 0.50).

Carapace dark brown (Fig. 14C). *Chelicerae* dark brown, m*axillae* brown with white tips, grey hairs on inner margins. *Labium* dark brown, light at tip with black hairs. *Sternum* greyish brown. Abdomen oval, greyish brown. Venter and spinnerets dark greyish. Legs I more robust and darker than others, which are yellow and black. Spination of leg I: femur d2-1-1; tibia p0-2-0, r0-2-0; metatarsus v2-0-2. Palp: tibia short, subequal to half the length of the cymbium. Tibial apophysis triangular and very short, about 1/5 the length of the tibia (Fig. 13B). Tegulum large, nearly rectangular. Embolus short, broad with bifurcate tip (Fig. 13D).

**Female** (one of paratypes) very similar to the male. Total length 6.00, CL 3.00, CW 2.10, AL 3.00, AW 1.76. Eye measurements: AME 0.60, ALE 0.40, PME 0.09, PLE 0.27, AER 2.00, PER 1.84, EFL 1.60. Clypeus 0.20 high. Legs: I 4.96 (1.50, 1.00, 1.13, 0.80, 0.53); II 4.31 (1.41, 0.75, 0.90, 0.75, 0.50); III 5.62 (1.85, 0.94, 1.13, 1.00, 0.70); IV 5.38 (1.64, 0.71, 1.13, 1.20, 0.70).

Legs light brown. Spination of leg I: femur d2-1-1; tibia v2-2-2; metatarsus v2-0-2. Epigyne wider than long, with posterior projection and two pockets (Fig. 14A). Copulatory openings with two semicircular highly sclerotised lobes. Copulatory ducts broad, anteriorly broader, receptacles massive, slightly curved and closely spaced. Fertilisation ducts at the anterior part of the receptacles (Fig. 14B).

###### Distribution.

Known from several localities in Xishuangbanna.

#### Genus *Chinophrys* Zhang & Maddison, 2012

##### Chinophrys mengyangensis

Taxon classificationAnimaliaAraneaeSalticidae

Cao & Li sp. n.

http://zoobank.org/045A5227-44EB-4D6B-953E-DDEFB089A229

Figs 15 , 16 , 43

###### Type.

**Holotype** ♂: CHINA, Yunnan, Jinghong City, Mengyang Town, seasonal rainforest (22°09.765'N, 100°52.553'E, 862 m), 22 July 2012, Q. Zhao & Z. Chen leg. **Paratypes**: 1♂, same data as holotype; 1♀, CHINA, Yunnan, Mengla County, Menglun Town, 48 km landmark of Nature Reserve (21°38.853'N, 101°09.625'E, 1001 m), seasonal rainforest, 30 July 2012, Q. Zhao & Z. Chen leg.; 1♂1♀, CHINA, Yunnan, Mengla County, Menglun Town, 48 km landmark in Nature Reserve (21°58.704'N, 101°19.748'E, 1088 m), seasonal rainforest, 12 August 2011, Q. Zhao & Z. Chen leg.

###### Etymology.

The species name is derived from the name of type locality; adjective.

###### Diagnosis.

**Figure 15.**
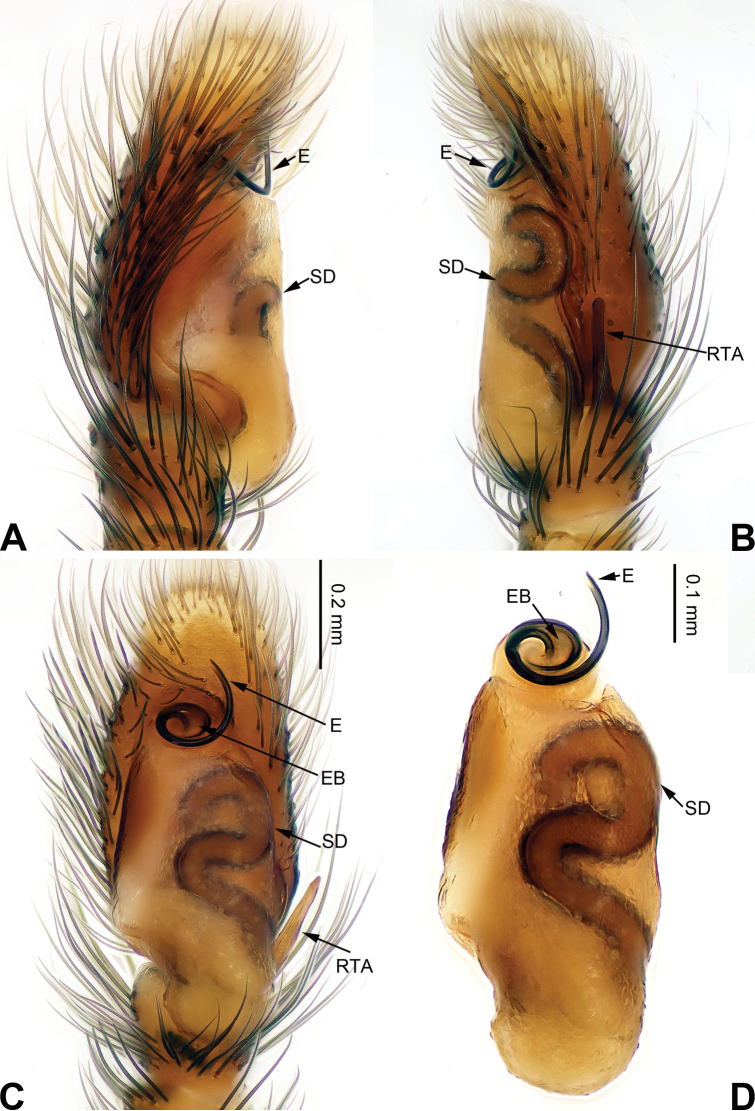
Palp of *Chinophrys mengyangensis* sp. n., male holotype. **A** prolateral **B** retrolateral **C** ventral **D** bulb, ventral. Scale bar equal for **A–C**.

**Figure 16.**
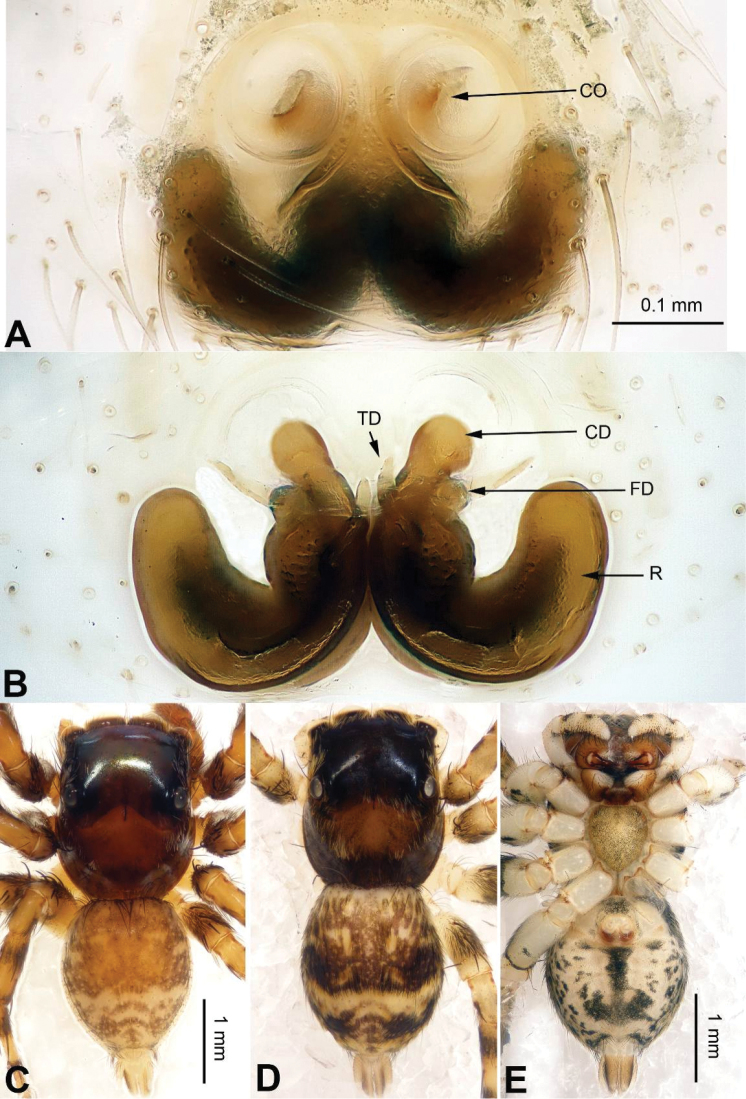
*Chinophrys mengyangensis* sp. n., female paratype and male holotype. **A** epigyne, ventral **B** vulva, dorsal **C** male habitus, dorsal **D** female habitus, dorsal **E** female habitus, ventral. Scale bars equal for **A** and **B**; equal for **D** and **E**.

Similar to *Chinophrys liujiapingensis* (Yang & Tang, 1997) (cf. Yang and Tang 1997: figs 6–10) in having a similar tegulum (Fig. 15D), but the embolus base is much wider. Compared to *Chinophrys pengi* (Zhang and Maddison 2012: figs 1–9), the tibial apophysis is located retrolaterally vs. dorso-retrolaterally. The epigyne of the new species resembles that of *Chinophrys pengi*, but the copulatory openings are different (the copulatory openings of the new species have fewer coils than in *Chinophrys pengi*) (Fig. 16A), and the receptacles lack spherical terminals vs. have spherical terminals in *Chinophrys pengi* (Fig. 16B).

###### Description.

**Male** (holotype). Total length 4.80, CL 2.25, CW 1.75, AL 2.55, AW 1.60. Eye measurements: AME 0.48, ALE 0.31, PME 0.05, PLE 0.29, AER 1.62, PER 1.70, EFL 1.44. Clypeus 0.10 high. Legs: I 4.40 (1.45, 0.70, 1.05, 0.70, 0.50); II 3.95 (1.30, 0.65, 0.80, 0.70, 0.50); III 4.60 (1.40, 0.50, 1.00, 1.00, 0.70); IV 4.85 (1.40, 0.60, 1.00, 1.10, 0.75).

Carapace dark brown, tegument iridescent with a few sparse colourless setae (Fig. 16C). Chelicerae, *maxillae* and labium greyish brown with white tips. Sternum greyish yellow. Abdomen oval and greyish brown. Venter and spinnerets grey. Legs light brown. Spination of leg I: femur d5-1-1; patella p0-1-0; tibia v2-2-2, p1-0-1, r1-0-1; metatarsus v2-0-2, p1-0-1 r1-0-1. Palp: tibia short, about 1/3 the length of the cymbium. Tibial apophysis straight, rod-like and subequal to the length of the tibia (Fig. 15B). Cymbium with long, dark brown bristles. Tegulum twice as long as wide. Seminal duct broad and coiled. Embolus a narrow helix (Fig. 15C).

**Female** (one of paratypes). Total length 4.73, CL 2.30, CW 2.03, AL 2.43, AW 1.94. Eye measurements: AME 0.60, ALE 0.33, PME 0.03, PLE 0.29, AER 1.79, PER 1.81, EFL 1.44. Clypeus 0.10 high. Legs: I 4.24 (1.39, 0.75, 0.90, 0.75, 0.45); II 4.04 (1.38, 0.70, 0.85, 0.66, 0.45); III 4.93(1.60, 0.80, 0.93, 1.05, 0.55); IV 5.32 (1.56, 0.75, 1.13, 1.25, 0.63).

Abdomen dark brown. Legs grey. Other characters similar to those of male. Epigyne: Copulatory ducts stout, receptacles kidney-shaped, with anteriorly bent translucent ducts (Fig. 16B). Fertilisation ducts located at the anterior part of the receptacles (Fig. 16B).

###### Distribution.

Known from several localities in Xishuangbanna.

#### Genus *Cocalus* C.L. Koch, 1846

##### Cocalus menglaensis

Taxon classificationAnimaliaAraneaeSalticidae

Cao & Li sp. n.

http://zoobank.org/A97C770D-4BF5-49DF-947E-F24ED92C29FE

Figs 17 , 18 , 43

###### Type.

**Holotype** ♂: CHINA, Yunnan, Mengla County, Xiaolongha Village, Xishuangbanna Nature Reserve, Biological diversity corridor (21°24.330'N, 101°37.002'E, 801 m), secondary forest, 30 June 2012, Q. Zhao & Z. Chen leg. **Paratypes**: 1♀, CHINA, Yunnan, Mengla County, Xiaolongha Village, Xishuangbanna Nature Reserve, Biological Diversity Corridor (21°24.265'N, 101°37.300'E, 653 m), seasonal rainforest, 27 June 2012, Q. Zhao & Z. Chen leg.; 1♀, CHINA, Yunnan, Mengla County, Menglun Town, 48 km landmark in Nature Reserve (21°58.704'N, 101°19.748'E, 1088 m), seasonal rainforest, 12 August 2011, Q. Zhao & Z. Chen leg.

###### Etymology.

The species name is derived from the name of the type locality; adjective.

###### Diagnosis.

**Figure 17.**
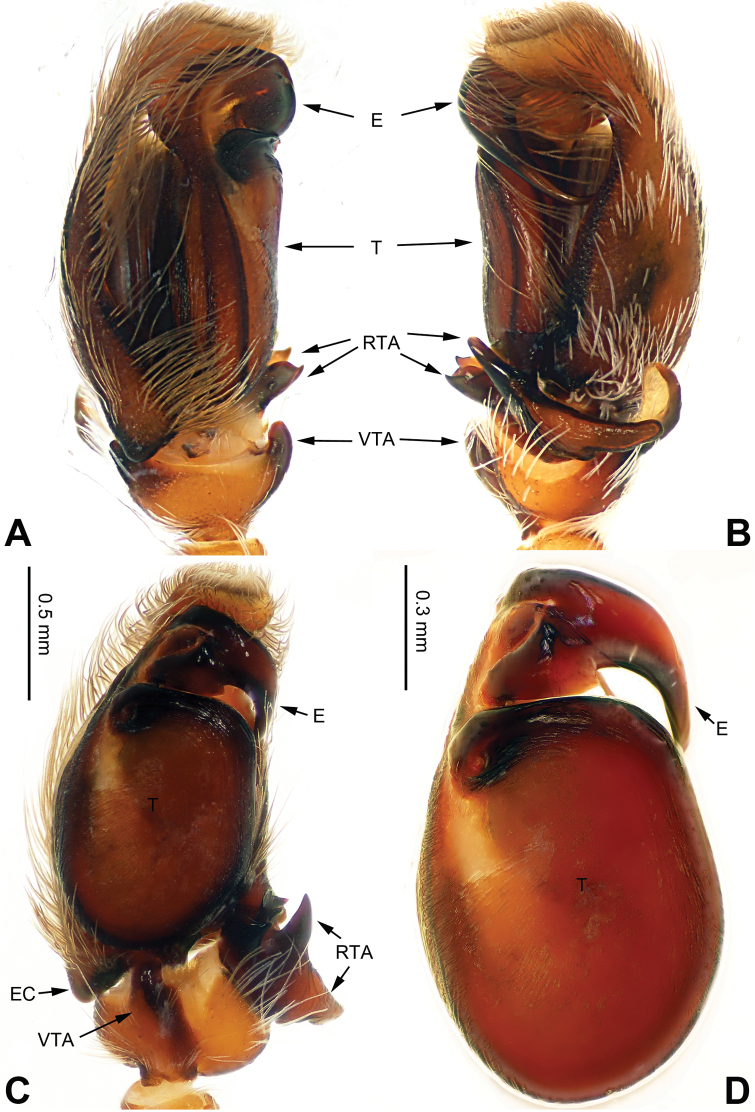
Palp of *Cocalus menglaensis* sp. n., male holotype. **A** prolateral **B** retrolateral **C** ventral **D** bulb, ventral. Scale bar equal for **A–C**.

**Figure 18.**
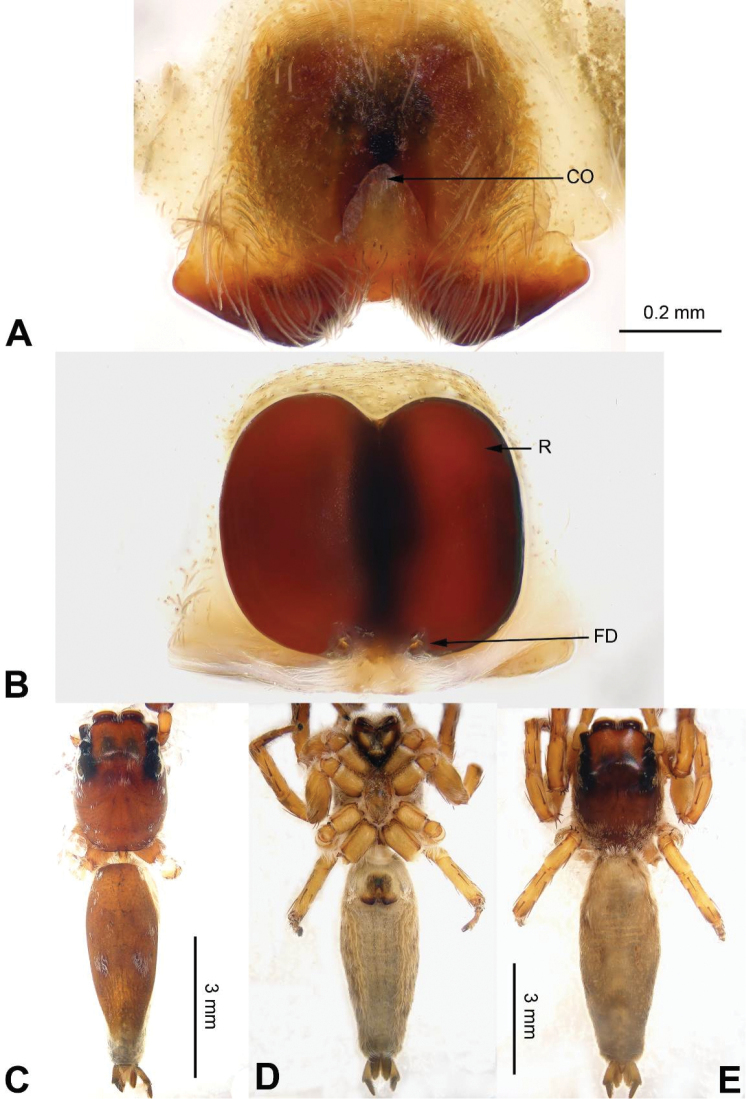
*Cocalus menglaensis* sp. n., female paratype and male holotype. **A** epigyne, ventral **B** vulva, dorsal **C** male habitus, dorsal **D** female habitus, dorsal **E** female habitus, ventral. Scale bars equal for **A** and **B**; equal for **D** and **E**.

Similar to *Cocalus gibbosus* Wanless, 1981 (see Wanless 1981: fig. 4A–D) by the shape of tegulum and embolus (Fig. 17C–D), but different in the shape of RTA. Female copulatory openings (Fig. 18A) resemble *Cocalus murinus* Simon, 1899 (see Wanless, 1981: 256, fig. 3A–E), but the epigynal plate has two rectangular and strongly sclerotised posterior projections (Fig. 18A)

###### Description.

**Male** (holotype). Total length 8.75, CL 3.75, CW 2.25, AL 5.00, AW 1.50. Eye measurements: AME 0.60, ALE 0.25, PME 0.22, PLE 0.26, AER 1.86, PER 2.00, EFL 1.74. Clypeus 0.31 high. Legs: I 8.10 (2.10, 1.25, 2.00, 1.75, 1.00); II 6.80 (2.00, 1.00, 1.50, 1.50, 0.80); III 7.27 (1.95, 1.20, 1.75, 1.57, 0.80); IV 8.95 (2.50, 1.15, 2.00, 2.20, 1.10).

Carapace dark brown with short recumbent and white setae (Fig. 18C). *Eyes* surrounded with black except AME. Clypeus covered with dark grey hairs. *Chelicerae* dark brown, sparsely covered with fine black hairs. *Maxillae* brownish with dull white tips and dark grey hairs on inner margins. *Labium* dark brown, tip dull white. *Sternum* grey-brown, with few black hairs. Abdomen dorsally orange-brown to greyish brown, clothed in short recumbent white hairs. Venter dark greyish. Spinnerets dark brown. Legs generally amber. Spination of leg I: femur d4-1-1; patella p0-1-0; tibia v2-0-2, p1-1-1, r1-1-1; metatarsus v0-2-0, p0-1-0 r0-1-0 d2-1-2. Palp: densely covered by hairs. Cymbium with posterior triangular extension prolaterally (Fig. 17C). Tegulum ovoid, with tegular furrow and dark peripheral seminal duct. Embolus stout, bending ventrally (Fig. 17C).

**Female** (one of the paratypes). Total length 10.67, PL 4.00, PW 2.67, OL 6.67, OW 2.19. Eye measurements: AME 0.60, ALE 0.30, PME 0.25, PLE 0.35, AER 2.00, PER 2.45, EFL 1.74. Clypeus 0.20 high. Legs: I 8.08 (2.23, 1.53, 2.05, 1.47, 0.80); II 7.10 (2.00, 1.36, 1.74, 1.25, 0.75); III 7.27 (2.10, 1.20, 1.66, 1.41, 0.90); IV 9.91 (2.66, 1.40, 2.25, 2.40, 1.20).

Abdomen dorsally greyish. Spination of leg I: femur d4-1-1; patella p0-1-0, r0-1-0; tibia v2-2-2, p1-0-1, r1-0-1, d1-1-1; metatarsus v0-2-0, d2-0-2. Other characters similar to those of male. Epigyne: dark amber clothed in creamy hairs. Epigyne with two rectangular and strongly sclerotised posterior projections (Fig. 18A). Receptacles massive and phaseoliform, fertilisation ducts located at the posterior part of the receptacles (Fig. 18B).

###### Distribution.

Known from several localities in Xishuangbanna.

#### Genus *Cosmophasis* Simon, 1901

##### Cosmophasis xiaolonghaensis

Taxon classificationAnimaliaAraneaeSalticidae

Cao & Li sp. n.

http://zoobank.org/D0DE08D5-D373-49A7-A206-5CA130E41DE9

Figs 19 , 20 , 43

###### Type.

**Holotype** ♂: CHINA, Yunnan, Mengla County, Xiaolongha Village, Xishuangbanna Nature Reserve: Biological diversity corridor (21°24.798'N, 101°37.880'E, 693 m), seasonal rainforest, 28 June 2012, Q. Zhao & Z. Chen leg. **Paratypes**: 1♂1♀, same data as holotype; 1♀, CHINA, Yunnan, Mengla County, Xiaolongha Village, Xishuangbanna Nature Reserve: Biological diversity corridor (21°24.230'N, 101°36.262'E, 715 m), seasonal rainforest, 4 June 2012, Q. Zhao & Z. Chen leg.

###### Etymology.

The species name is derived from the name of the type locality; adjective.

###### Diagnosis.

**Figure 19.**
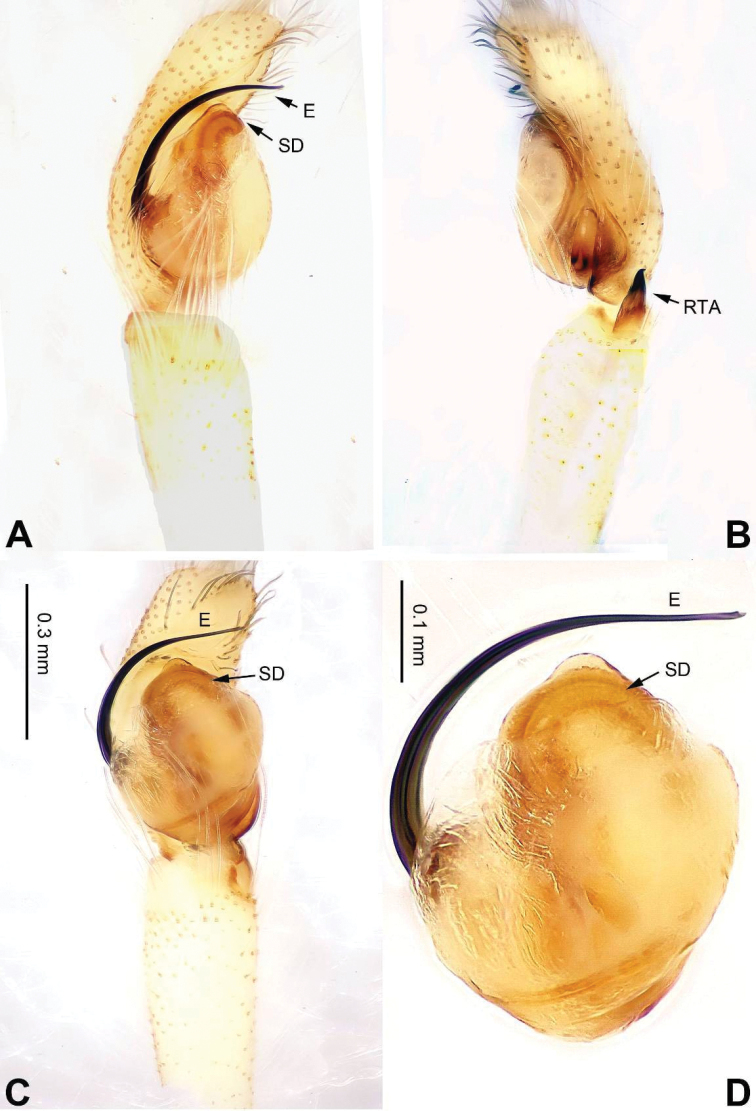
Palp of *Cosmophasis xiaolonghaensis* sp. n., male holotype. **A** prolateral **B** retrolateral **C** ventral **D** bulb, ventral. Scale bar equal for **A–C**.

**Figure 20.**
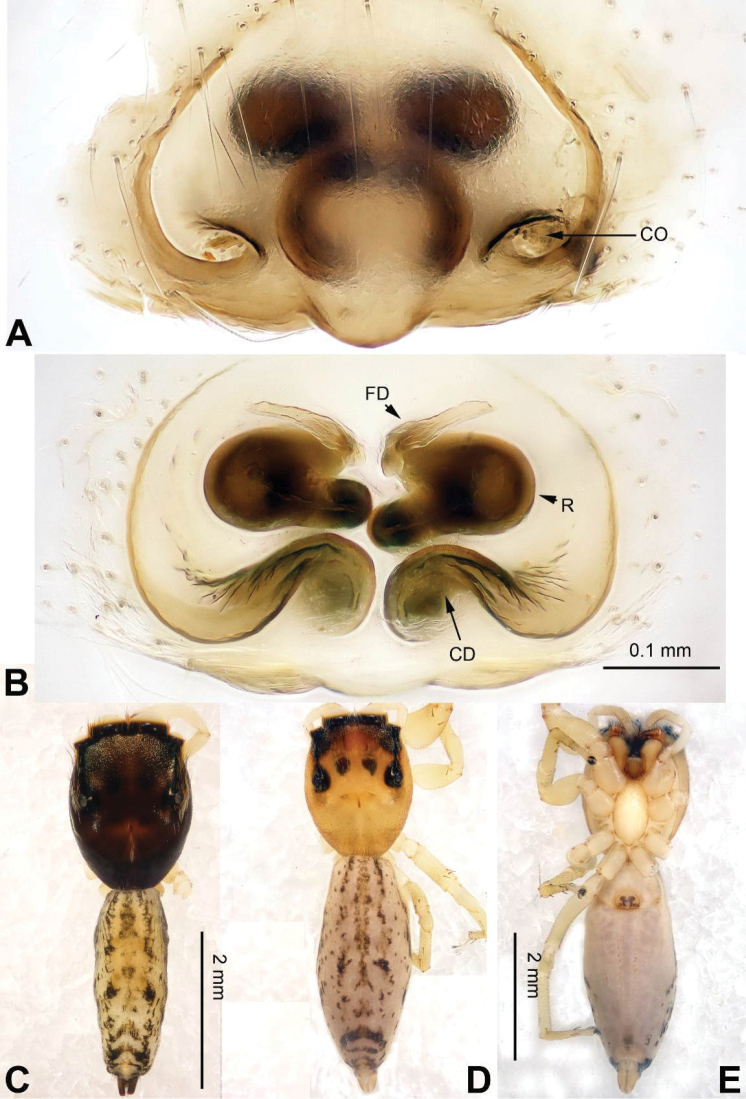
*Cosmophasis xiaolonghaensis* sp. n., female paratype and male holotype. **A** epigyne, ventral **B** vulva, dorsal **C** male habitus, dorsal **D** female habitus, dorsal **E** female habitus, ventral. Scale bars equal for **A** and **B**; equal for **D** and **E**.

Similar to *Cosmophasis courti* Żabka & Waldock, 2012 (Żabka and Waldock 2012: figs 52A–H, 53A–D), but tibia is subequal to cymbium (Fig. 19A–B) vs. twice as long in *Cosmophasis courti*, RTA with one small apical hook (Fig. 19B), lacking in *Cosmophasis courti*. Copulatory openings widely separated (Fig. 20A), about three diameters vs. only 1/4 the diameter of a copulatory opening.

###### Description.

**Male** (holotype). Total length 5.20, CL 2.30, CW 1.72, AL 2.90, AW 1.05. Eye measurements: AME 0.55, ALE 0.24, PME 0.05, PLE 0.20, AER 1.59, PER 1.59, EFL 1.44. Clypeus 0.09 high. Legs: I 4.34 (1.34, 0.94, 1.00, 0.56, 0.50); II 3.64 (1.13, 0.70, 0.76, 0.58, 0.47); III 3.84 (1.30, 0.55, 0.71, 0.80, 0.48); IV 4.31 (1.41, 0.56, 0.70, 1.06, 0.50).

Carapace dark brown (Fig. 20C). *Chelicerae* dark brown, sparsely covered with fine grey hairs. *Maxillae* brown with light tips and grey hairs on inner margins. *Labium* brown, tip with black hairs. Abdomen light, with irregular black patches. Venter and spinnerets dark grey. Legs I more robust and darker than others. Spination of leg I: femur d2-1-0; tibia v2-2-2; metatarsus v2-0-2. Palpal tibia white, subequal to the cymbium. The apophysis short, about 1/4 the length of the cymbium, with a small apical hook (Fig. 19B). Embolus starting at the prolateral part of the bulb, bent (Fig. 19C).

**Female** (same locality of holotype). Total length 6.46, CL 2.66, CW 1.90, AL 3.80, AW 1.64. Eye measurements: AME 0.60, ALE 0.22, PME 0.06, PLE 0.22, AER 1.59, PER 1.59, EFL 1.44. Clypeus 0.08 high. Legs: I 4.34 (1.34, 0.94, 1.00, 0.56, 0.50); II 3.64 (1.13, 0.70, 0.76, 0.58, 0.47); III 3.84 (1.30, 0.55, 0.71, 0.80, 0.48); IV 4.31 (1.41, 0.56, 0.70, 1.06, 0.50).

Carapace yellow (Fig. 20D). Abdominal venter grey-white. Legs yellowish. Spination of leg I: femur d2-1-0; tibia v2-2-2; metatarsus v2-0-2. Other characters similar to those of male. Epigynal plate weakly sclerotized. Copulatory opening grooves with sclerotised edges, openings widely separated (about three diameters). Posterior margin projecting in midline (Fig. 20A). Copulatory ducts broad, located posteriorly. Receptacles round, strongly sclerotised. Fertilisation ducts elongate, located at the anterior part of the receptacles (Fig. 20B).

###### Distribution.

Known from several localities in Xishuangbanna.

#### Genus *Cytaea* Keyserling, 1882

##### Cytaea yunnanensis

Taxon classificationAnimaliaAraneaeSalticidae

Cao & Li sp. n.

http://zoobank.org/0A78F858-FEC9-41E2-B86F-95FC8B1714ED

Figs 21 , 22 , 43

###### Type.

**Holotype** ♂: CHINA, Yunnan, Mengla County, Menglun Town, Menglun Nature Reserve (21°57.669'N, 101°11.893'E, 790 m), 23 April 2007, G. Zheng leg.

###### Etymology.

The species name derived from the name of the type locality; adjective.

###### Diagnosis.

**Figure 21.**
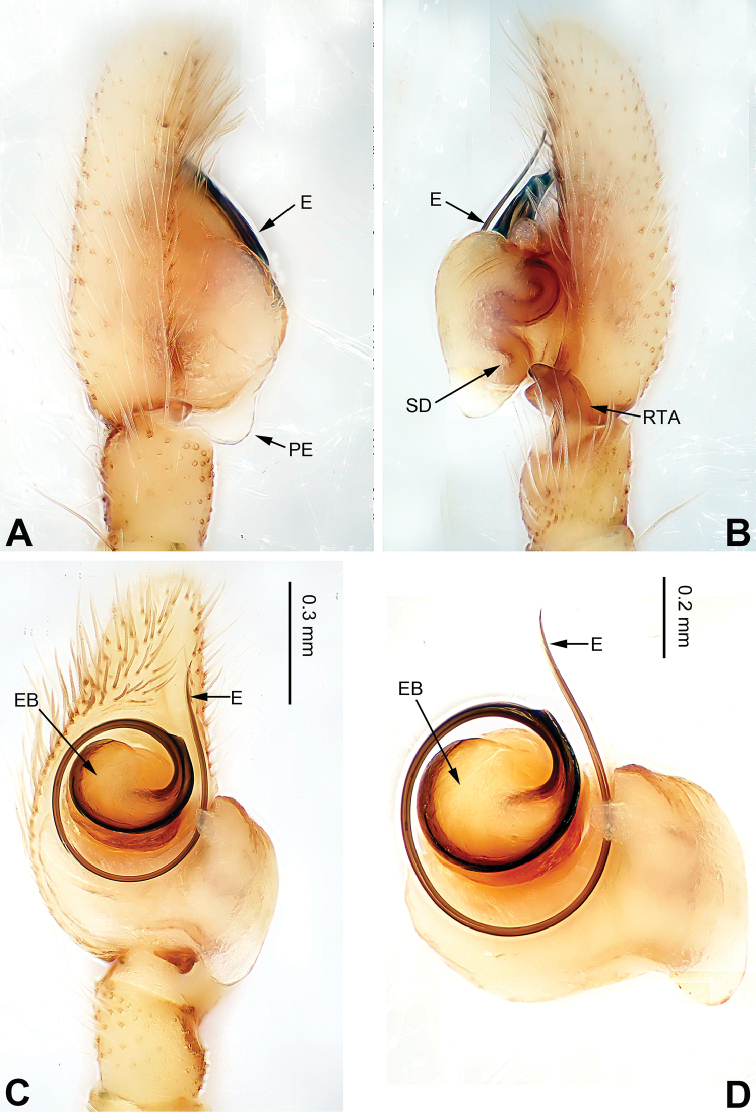
Palp of *Cytaea yunnanensis* sp. n., male holotype. **A** prolateral **B** retrolateral **C** ventral **D** bulb, ventral. Scale bar equal for **A–C**.

Differs from all known congeners by the shape of the tegulum (prolateral large bulge and posterior translucent extension) (Fig. 21).

###### Description.

**Male** (holotype). Total length 5.75, CL 2.75, CW 2.05, AL 3.00, AW 1.75. Eye measurements: AME 0.55, ALE 0.36, PME 0.10, PLE 0.36, AER 1.90, PER 1.80, EFL 1.50. Clypeus 0.13 high. Legs: I 4.76 (1.38, 0.75, 1.13, 0.90, 0.60); II 5.56 (1.63, 0.88, 1.20, 0.95, 0.90); III missing; IV 5.63 (1.63, 0.75, 1.30, 1.20, 0.75).

**Figure 22.**
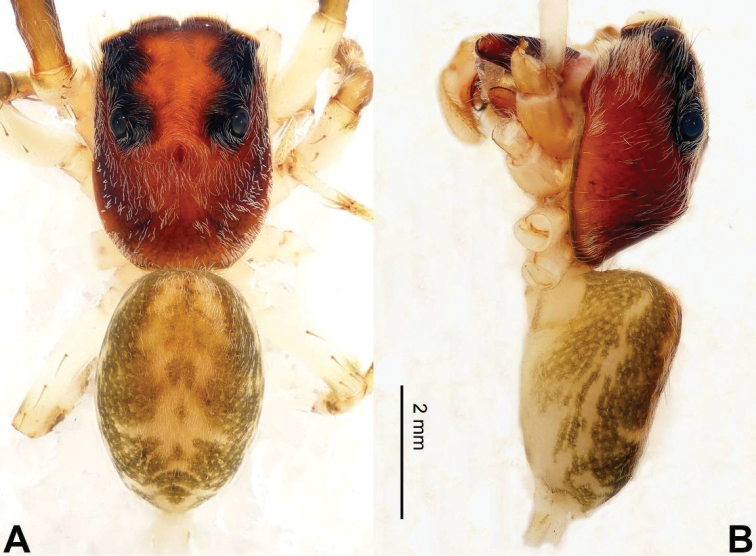
Habitus of *Cytaea yunnanensis* sp. n., male holotype. **A** dorsal **B** lateral.

Carapace brown, covered with dense, white setae (Fig. 22A). Lateral *eyes* surrounded with black. *Chelicerae* dark orange-brown. *Maxillae* brownish, with dull white tips. *Labium* dark brown, tip dull white. *Sternum* light brown. Abdomen oval, dorsally greyish brown, narrower than carapace. Venter greyish. Spinnerets light greyish. Legs whitish. Spination of leg I: femur d2-1-1; tibia v2-2-2, p1-0-1; metatarsus v2-0-2, p0-1-0. Palpal tibia short, about 1/4 length of cymbium. The tibial apophysis subequal to the tibia, with a slightly bent tip (Fig. 21B). Cymbium whitish. Tegulum with meandering seminal duct and prominent prolateral bulge. Embolus with circular basal pad (Fig. 21C).

**Female.** Unknown.

###### Distribution.

Known only from the type locality.

#### Genus *Gedea* Simon, 1876

##### Gedea pinguis

Taxon classificationAnimaliaAraneaeSalticidae

Cao & Li sp. n.

http://zoobank.org/8B648ACB-2493-4AAE-B632-C88649335E2E

Figs 23 , 43

###### Type.

**Holotype** ♂: CHINA, Yunnan, Mengla County, Menglun Town, Xishuangbanna Nature Reserve, G213 road, Banyan tree (21°54.089'N, 101°17.024'E, 579 m), 28 November 2009, G. Tang & Z. Yao leg.

###### Etymology.

From Latin *pinguis* (fat), in reference to the shape of the palp; adjective.

###### Diagnosis.

**Figure 23.**
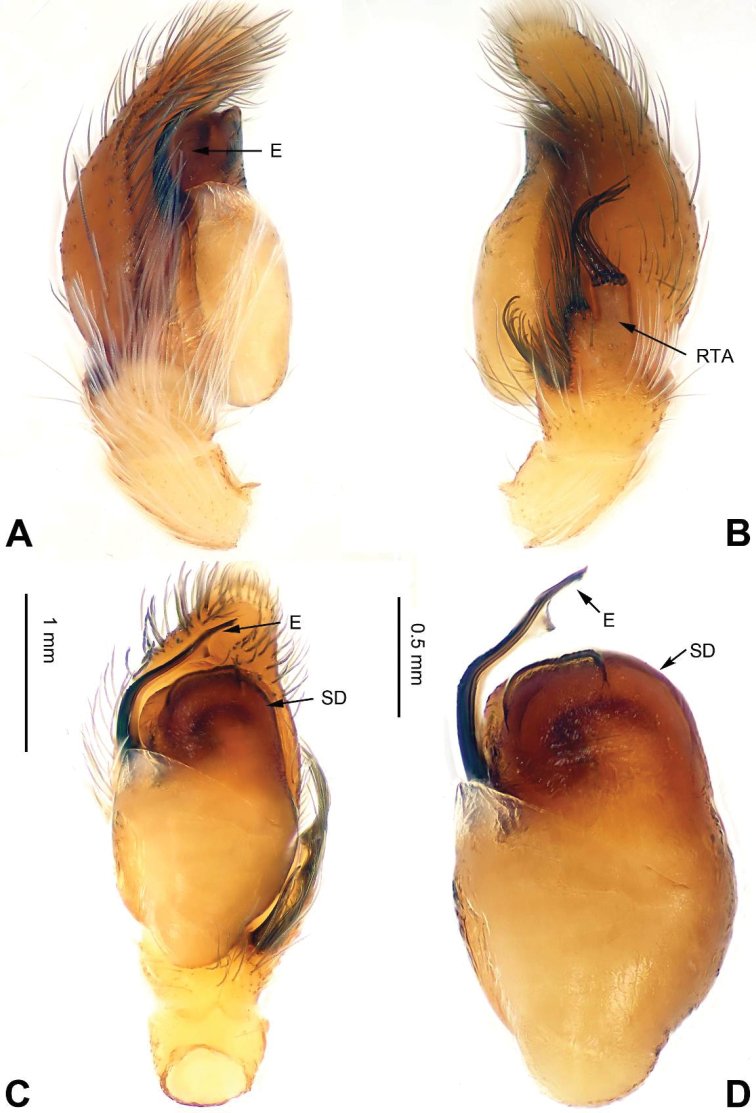
Palp of *Gedea pinguis* sp. n., male holotype. **A** prolateral **B** retrolateral **C** ventral **D** bulb, ventral. Scale bar equal for **A–C**.

Similar to *Gedea tibialis* Żabka, 1985 (see Żabka 1985: figs 263–267), but the cymbium is shorter (about 1/3 the length of the bulb in the new species vs. nearly equal in *Gedea tibialis*), embolus base with membrane (without in *Gedea tibialis*) (Fig. 23C–D), tibial apophysis with two branches, both with clusters of long apical bristles (only one without bristles in *Gedea tibialis*) (Fig. 23B).

###### Description.

**Male** (holotype). In poor condition. Precise description of the habitus unavailable. Palpal tibia short, subequal to the tip of the cymbium, about 1/3 the length of the bulb. Tibial apophysis with two branches, each with clusters of long apical bristles (Fig. 23B). Tegulum with posterior lobe, and the width about 2/3 of the length. Embolus base hidden by the fold of the membranous structure of the tegulum (Fig. 23C). Embolus accompanied by a membrane, membrane with a triangular outgrowth (Fig. 23D).

**Female.** Unknown.

###### Distribution.

Known only from the type locality.

#### Genus *Gelotia* Thorell, 1890

##### Gelotia zhengi

Taxon classificationAnimaliaAraneaeSalticidae

Cao & Li sp. n.

http://zoobank.org/8C8AD320-9364-481E-B657-B707C24B3CD8

Figs 24 , 25 , 43

###### Type.

**Holotype** ♂: CHINA, Yunnan, Mengla County, Menglun Town, Xishuangbanna Tropical Botanical Garden, Chinese Academy of Sciences: Lvshilin (21°54.705'N, 101°16.898'E, 656 m), 11 November 2009, G. Zheng leg.

###### Etymology.

The specific is named in honour of the collector Guo Zheng from Shenyang Normal University; noun (name) in genitive case.

###### Diagnosis.

**Figure 24.**
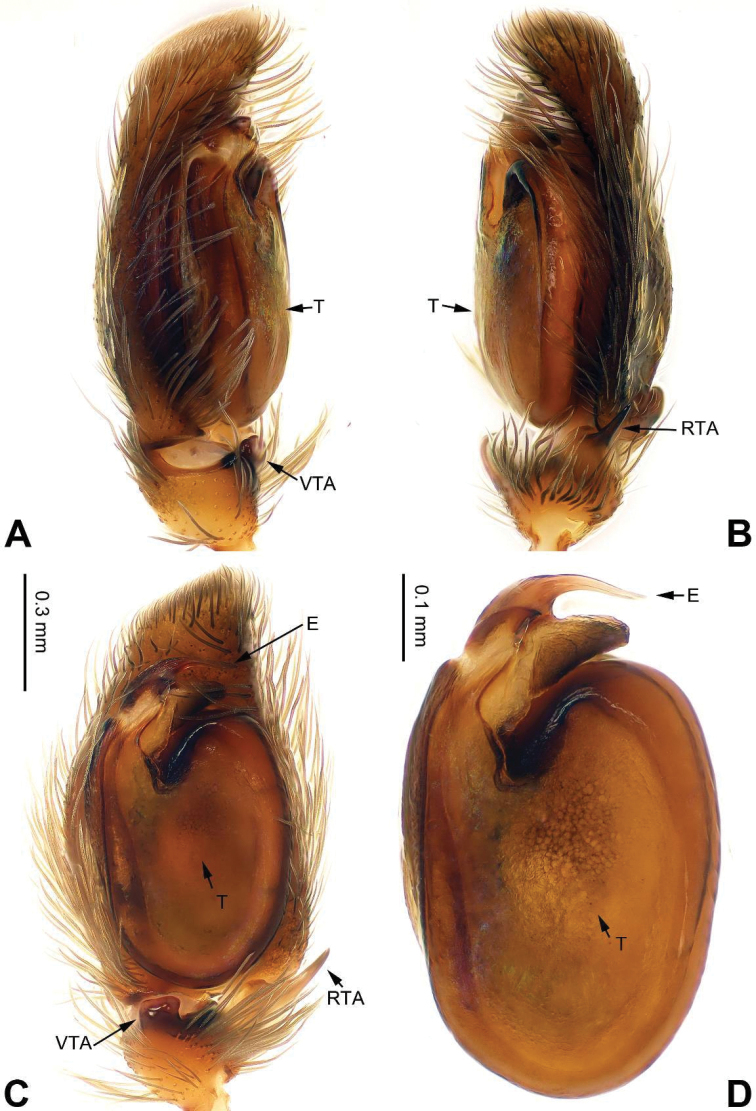
Palp of *Gelotia zhengi* sp. n., male holotype. **A** prolateral **B** retrolateral **C** ventral **D** bulb, ventral. Scale bar equal for **A–C**.

Differs from the similar *Gelotia bouchardi* (Simon, 1903) (see Prószyński and Deeleman-Reinhold 2012: figs 47–50) by the shorter embolus (subequal to the tip of the cymbium vs. nearly twice as long in *Gelotia bouchardi*) (Fig. 24C) and the straight RTA (Fig. 24B) vs. bent in *Gelotia bouchardi*.

###### Description.

**Male** (holotype). Total length 5.90, CL 2.75, CW 1.80, AL 3.15, AW 1.60. Eye measurements: AME 0.50, ALE 0.28, PME 0.15, PLE 0.20, AER 1.68, PER 1.68, EFL 1.40. Clypeus 0.25 high. Legs: I 8.15 (2.05, 0.85, 2.10, 2.10, 1.05); II 6.85 (1.90, 0.80, 1.75, 1.55, 0.85); III 6.30 (1.75, 0.70, 1.45, 1.55, 0.85); IV 8.30 (2.15, 0.85, 1.90, 2.35, 1.05).

**Figure 25.**
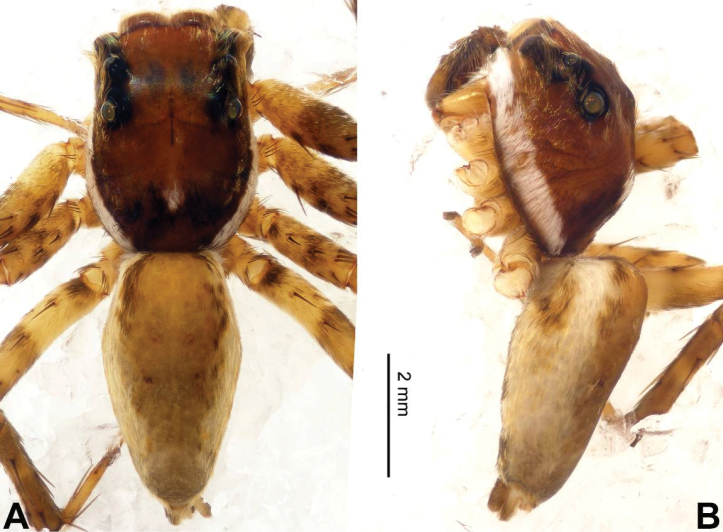
Habitus of *Gelotia zhengi* sp. n., male holotype. **A** dorsal **B** lateral.

Carapace dark brown (Fig. 25A), sides and clypeus margins encircled with a wide band of white hairs. Chelicerae light brown, inner margin with greyish brown setae. *Maxillae brown, tips with grey hairs.* Labium dark brown, light tip with grey hairs. Sternum greyish brown. Abdomen oval, greyish brown. Venter and spinnerets dark greyish. Legs brown, slender. Spination of leg I: femur d4-1-1; patella p0-1-0, r0-1-0; tibia v2-2-2, p1-0-1, d1-1-1, r1-0-1; metatarsus v2-0-2, p1-1-1, d2-0-2, r1-1-1. Palpal tibia short, about 1/5 the length of the cymbium. The ventral tibial apophysis short and obtuse, retrolateral apophysis broad at the base and sharp apically (Fig. 24B). Cymbium flattened and semilunar. Tegulum subovoid with peripheral seminal duct. Embolus base with one stout lobe (Fig. 24D).

**Female.** Unknown.

###### Distribution.

Known only from the type locality.

#### Genus *Icius* Simon, 1876

##### Icius bamboo

Taxon classificationAnimaliaAraneaeSalticidae

Cao & Li sp. n.

http://zoobank.org/71D1D871-F4BE-434B-8956-B0CC0CB4CB33

Figs 26 , 27 , 43

###### Type.

**Holotype** ♂: CHINA, Yunnan, Mengla County, Menglun Town, Bamboo plantation, G213 (21°53.646'N, 101°16.975'E, 589 m), 26 November 2009, G. Tang & Z. Yao leg.

###### Etymology.

The species was collected from a bamboo plantation; noun.

###### Diagnosis.

**Figure 26.**
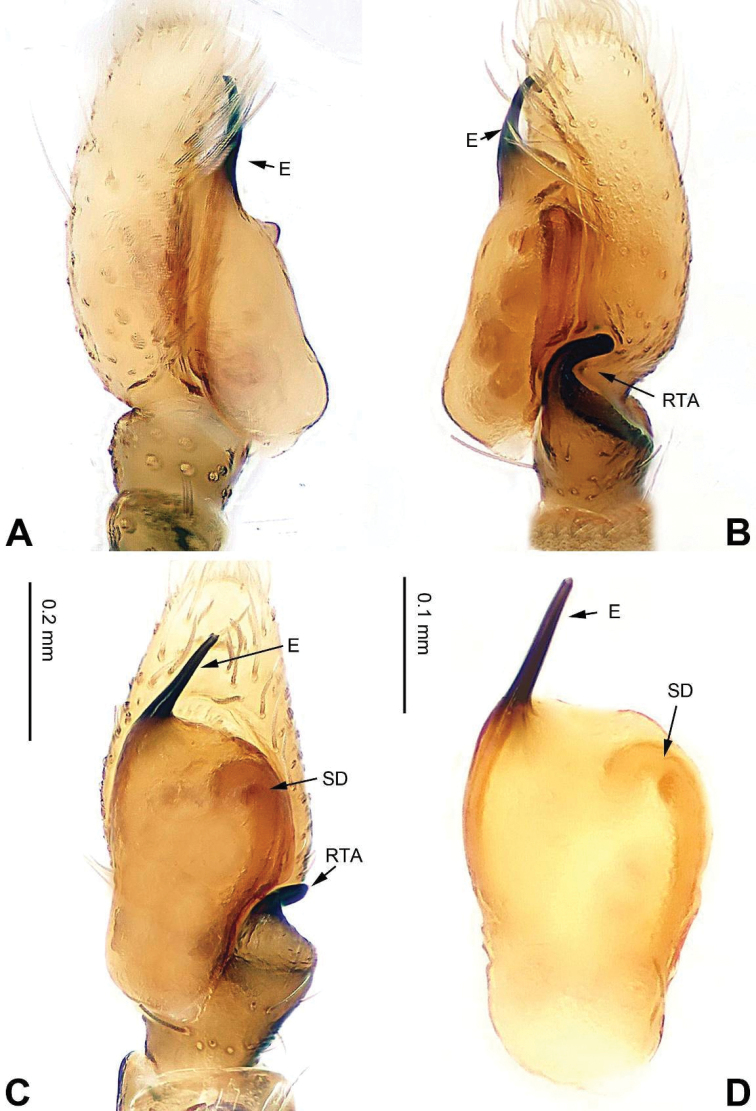
Palp of *Icius bamboo* sp. n., male holotype. **A** prolateral **B** retrolateral **C** ventral **D** bulb, ventral. Scale bars: equal for **A, B** and **C**.

The male resembles *Icius hamatus* (see Andreeva et al. 1984: figs 1–4), but the embolus is straight and digitiform (Fig. 26D) vs. bent and needle-like; the RTA is entire (unbranched) and bent dorsally (Fig. 26B) vs. with two branches and nearly triangular apophyses in *Icius hamatus*.

###### Description.

**Male** (holotype). Total length 4.23, CL 1.53, CW 0.90, AL 1.70, AW 0.80. Eye measurements: AME 0.31, ALE 0.15, PME 0.03, PLE 0.16, AER 1.00, PER 1.10, EFL 0.81. Clypeus 0.05 high. Legs: I 2.05 (0.65, 0.39, 0.49, 0.30, 0.22); II 1.70 (0.50, 0.30, 0.38, 0.27, 0.25); III 1.81 (0.56, 0.25, 0.40, 0.35, 0.25); IV 2.25 (0. 75, 0.32, 0.46, 0.44, 0.28).

**Figure 27.**
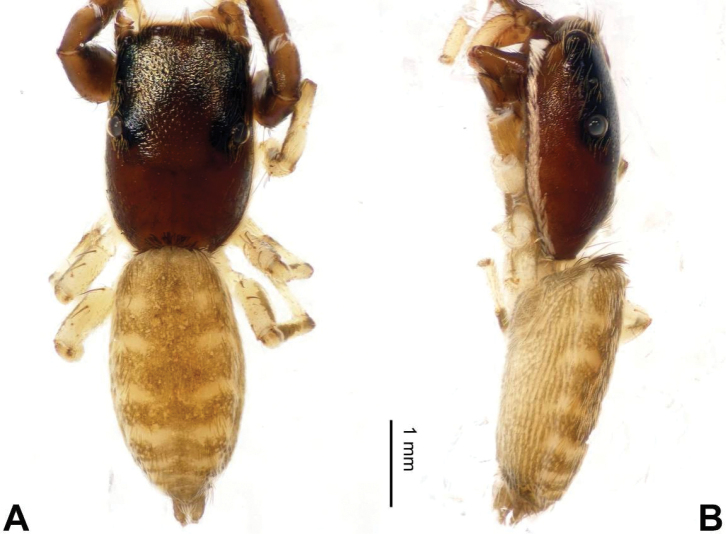
Habitus of *Icius bamboo* sp. n., male holotype. **A** dorsal **B** lateral.

Carapace dark brown (Fig. 27A). Sides and clypeus margins with a strip of white hairs. Chelicerae dark brown. *Maxillae* greyish yellow, inner margin with dense setae. Labium brown, tip with greyish hairs. Sternum greyish brown. Abdomen oval, with transverse alternating dark and light stripes. Venter and spinnerets grey. Legs I more robust and darker than other legs, which are yellowish. Spination of leg I: femur d4-1-1; tibia v2-0-2; metatarsus v2-0-2. Palpal tibia short, about 1/3 the length of cymbium, RTA bent, strong and with blunt tip (Fig. 26B). Bulb about twice as long as wide. Seminal duct encircling tegulum retrolaterally. Embolus short, digitiform, (Fig. 26D).

**Female.** Unknown.

###### Distribution.

Known only from the type locality.

#### Genus *Nannenus* Simon, 1902

##### Nannenus menghaiensis

Taxon classificationAnimaliaAraneaeSalticidae

Cao & Li sp. n.

http://zoobank.org/BF65EB9F-B57D-48B7-818C-F8A7FE33DC53

Figs 28 , 29 , 43

###### Type.

**Holotype** ♂: CHINA, Yunnan, Jinghong City, Menghai County, Menghai Village (22°01.702'N, 100°23.700'E, 1188 m), secondary forest, 28 July 2012, Q. Zhao & Z. Chen leg. **Paratypes**: 1♀, same data as holotype; 1♀, CHINA, Yunnan, Mengla County, Xiaolongha Village, Xishuangbanna Nature Reserve, Biological diversity corridor (21°24.213'N, 101°376.995'E, 834 m), seasonal rainforest, 3 June 2012, Q. Zhao & Z. Chen leg.

###### Etymology.

The species name is derived from the name of the type locality; adjective.

###### Diagnosis.

**Figure 28.**
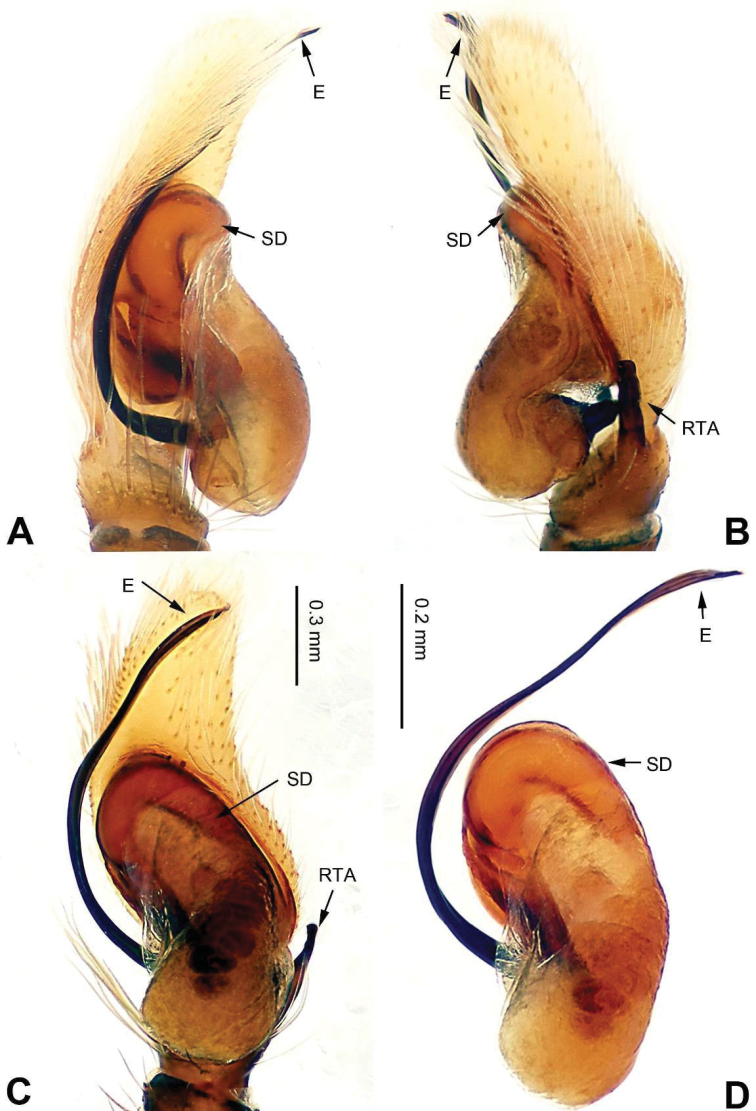
Palp of *Nannenus menghaiensis* sp. n., male holotype. **A** prolateral **B** retrolateral **C** ventral **D** bulb, ventral. Scale bar equal for **A–C**.

**Figure 29.**
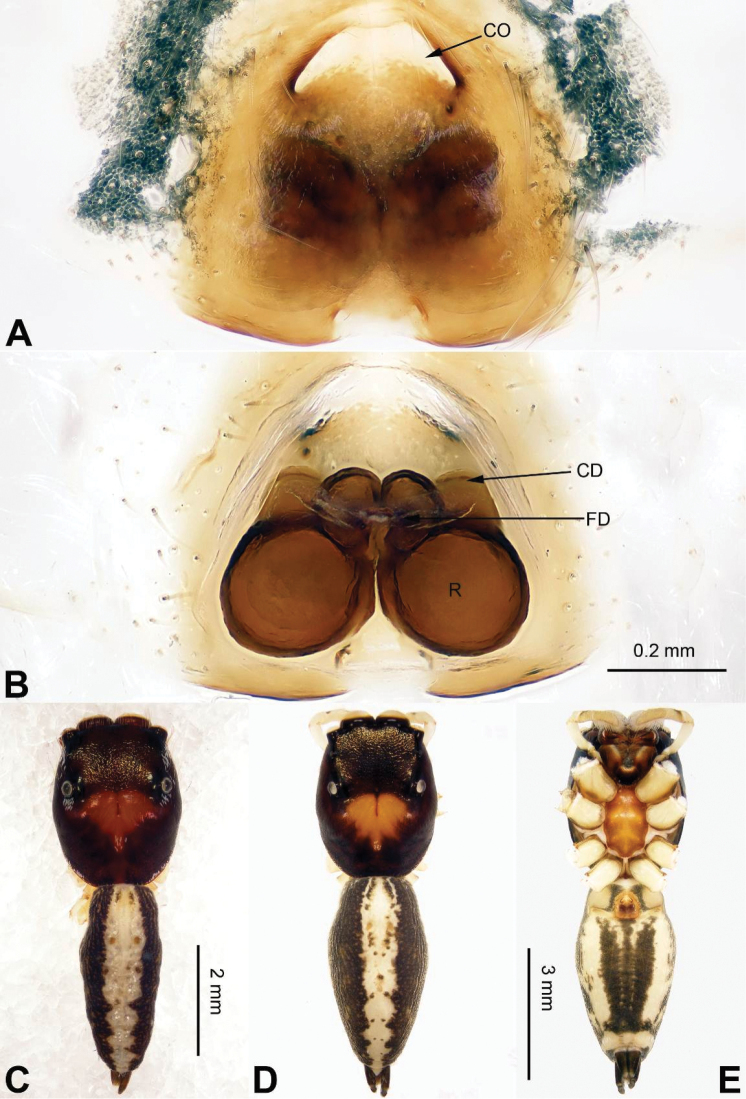
*Nannenus menghaiensis* sp. n., female paratype and male holotype. **A** epigyne, ventral **B** vulva, dorsal **C** male habitus, dorsal **D** female habitus, dorsal **E** female habitus, ventral. Scale bars equal for **A** and **B**; equal for **D** and **E**.

The male resembles that of *Nannenus maughami* Prószyński & Deeleman-Reinhold, 2012 (see Prószyński and Deeleman-Reinhold 2012: figs 97–99), but the RTA is straight and shorter, about 1/4 the length of the cymbium (Fig. 28B) vs. apically bent in *Nannenus maughami*. The female can be distinguished from other congeners by the boomerang-like copulatory openings (Fig. 29A).

###### Description.

**Male** (holotype). Total length 7.25, CL 3.50, CW 2.43, AL 3.75, AW 1.50. Eye measurements: AME 0.70, ALE 0.32, PME 0.15, PLE 0.25, AER 2.10, PER 2.15, EFL 1.80. Clypeus 0.25 high. Legs: I 6.00 (2.00, 1.10, 1.25, 0.90, 0.75); II 5.45 (1.65, 1.00, 1.20, 0.85, 0.75); III 5.48(1.70, 0.85, 1.00, 1.10, 0.83); IV 6.52 (1.95, 0.92, 1.50, 1.35, 0.80).

Carapace dark brown (Fig. 29C). Lateral *eyes* with surrounded with black. *Chelicerae* dark orange-brown, sparsely covered with fine grey hairs. *Maxillae* brownish with dull white tips and with grey hairs on the inner margins. *Labium* dark brown, with dull white tip and black hairs. *Sternum* orange-brown. Abdomen with central light stripe, black laterally. Venter dark greyish. Spinnerets black. Legs I more robust and darker than the other legs, which are yellow. Spination of leg I: femur d2-1-1; tibia v2-2-2; metatarsus v2-0-2. Palpal tibia short, about 1/5 the length of the cymbium, RTA straight, as long as tibia (Fig. 28B). Cymbium bent. Bulb elongate, set obliquely, with peripheral seminal duct. Embolus arising from basal-retrolateral part of the bulb, running parallel to bulb and extending to the tip of the cymbium (Fig. 28C).

**Female** (one of paratypes, same locality as holotype). Total length 9.26, CL 4.00, CW 2.56, AL 5.26, AW 2.38. Eye measurements: AME 0.79, ALE 0.39, PME 0.08, PLE 0.34, AER 2.28, PER 2.30, EFL 2.10. Clypeus 0.13 high. Legs: I 6.57 (2.15, 1.34, 1.48, 0.90, 0.70); II 6.02 (1.90, 1.20, 1.25, 0.95, 0.72); III 6.11 (1.90, 1.08, 1.00, 1.34, 0.79); IV 7.77 (2.28, 1.18, 1.72, 1.80, 0.79).

Spination of leg I: femur d0-1-0; tibia v2-2-2; metatarsus v2-0-2. Other characters similar to those of male. Epigyne with two rectangular posterior projections and central bulge formed by the copulatory ducts (Fig. 29A). Copulatory openings boomerang-like, located anteriorly. Copulatory ducts short, receptacles round, fertilisation ducts at the anterior part of the receptacles (Fig. 29B).

###### Distribution.

Known from several localities in Xishuangbanna.

#### Genus *Pancorius* Simon, 1902

##### Pancorius latus

Taxon classificationAnimaliaAraneaeSalticidae

Cao & Li sp. n.

http://zoobank.org/7DD1BEB1-7962-45C6-9AA9-709AEDABB168

Figs 30 , 31 , 43

###### Type.

**Holotype** ♂: CHINA, Yunnan, Mengla County, Menglun Town, *Paramichelia baillonii* plantation (21°54.200'N, 101°16.923'E, 608 m), 7 April 2007, leg. G. Zheng Guo.

###### Etymology.

From Latin *latus* (wide), in reference to the shape of the carapace; adjective.

###### Diagnosis.

**Figure 30.**
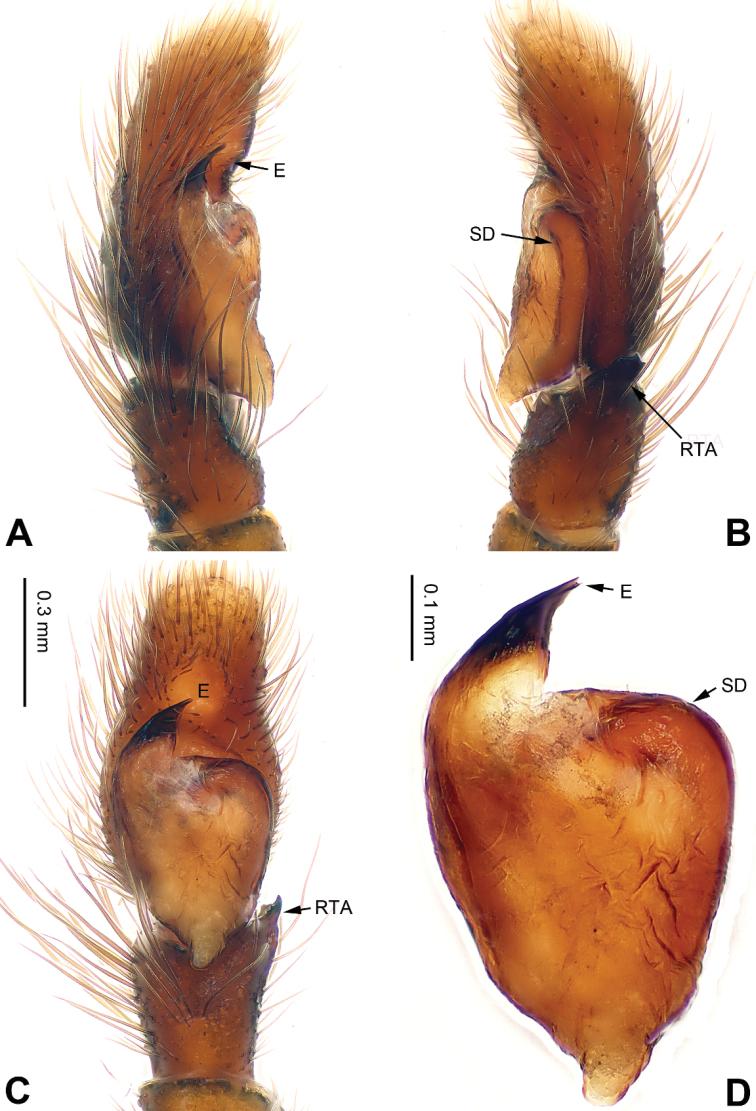
Palp of *Pancorius latus* sp. n., male holotype. **A** prolateral **B** retrolateral **C** ventral **D** bulb, ventral. Scale bar equal for **A–C**.

Similar to *Pancorius crassipes* (Karsch, 1881) (see Żabka 1997: figs 108–109), but bulb triangular (Fig. 30D) vs. oval, embolus beak-like with broad base (Fig. 30C) vs. needle-like; RTA rectangular with three little tips (Fig. 30B) vs. triangular in *Pancorius crassipes*.

###### Description.

**Male** (holotype). Total length 6.45, CL 3.20, CW 2.80, AL 3.25, AW 1.80. Eye measurements: AME 0.60, ALE 0.36, PME 0.07, PLE 0.36, AER 2.25, PER 2.20, EFL 1.90. Clypeus 0.31 high. Legs: I 8.40 (2.35, 1.25, 2.30, 1.60, 0.90); II 6.35 (2.00, 0.95, 1.45, 1.20, 0.75); III 6.80 (1.00, 1.30, 1.45, 0.95, 2.10); IV missing.

**Figure 31.**
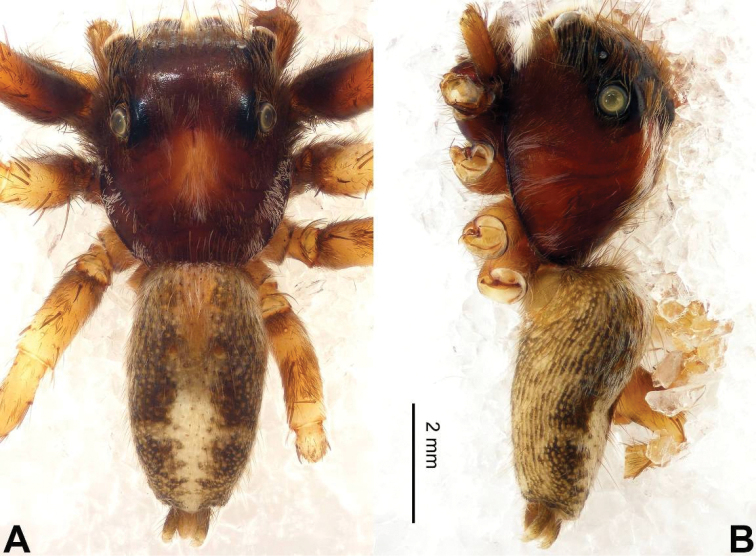
Habitus of *Pancorius latus* sp. n., male holotype. **A** dorsal **B** lateral.

Carapace dark brown, moderately high and slightly broadened posteriorly, with white or greyish hairs and lighter mediodorsally (Fig. 31A). Clypeus with dense white setae. Chelicerae dark brown, with greyish hairs. *Maxillae* and labium dark brown, tips with dark setae. Sternum light brown. Abdomen oval, generally grey. Venter and spinnerets greyish-= brown. Legs brown with hairs and spines. Spination of leg I: femur d3-1-1; patella p0-1-0; tibia v2-2-2, p1-0-1; metatarsus v2-0-2, p1-0-0. Palpal tibia about 1/2 the length of the cymbium. RTA rectangular, with three little apical tips (Fig. 30B). Bulb with posterior lobe. Embolus beak-like with broad base, subequal to the length of the RTA (Fig. 30C).

**Female.** Unknown.

###### Distribution.

Known from the type locality.

#### Genus *Phintella* Strand, 1906

##### Phintella lepidus

Taxon classificationAnimaliaAraneaeSalticidae

Cao & Li sp. n.

http://zoobank.org/D3974470-7A73-42D5-A2CC-0B3FBB801E8A

Figs 32 , 33 , 43

###### Type.

**Holotype** ♂: CHINA, Yunnan, Jinghong City, Mengyang Town (22°09.765'N, 100°52.553'E, 862 m), seasonal rainforest, 22 July 2012, Q. Zhao & Z. Chen leg. **Paratypes**: 1♂1♀, CHINA, Yunnan, Mengla County, Xiaolongha Village, Xishuangbanna Nature Reserve, Biological diversity corridor (21°24.192'N, 101°37.025'E, 657 m), seasonal rainforest, 29 July 2012, Q. Zhao & Z. Chen leg.

###### Etymology.

From Latin *lepidus* (nice), in reference to the body appearance; adjective.

###### Diagnosis.

**Figure 32.**
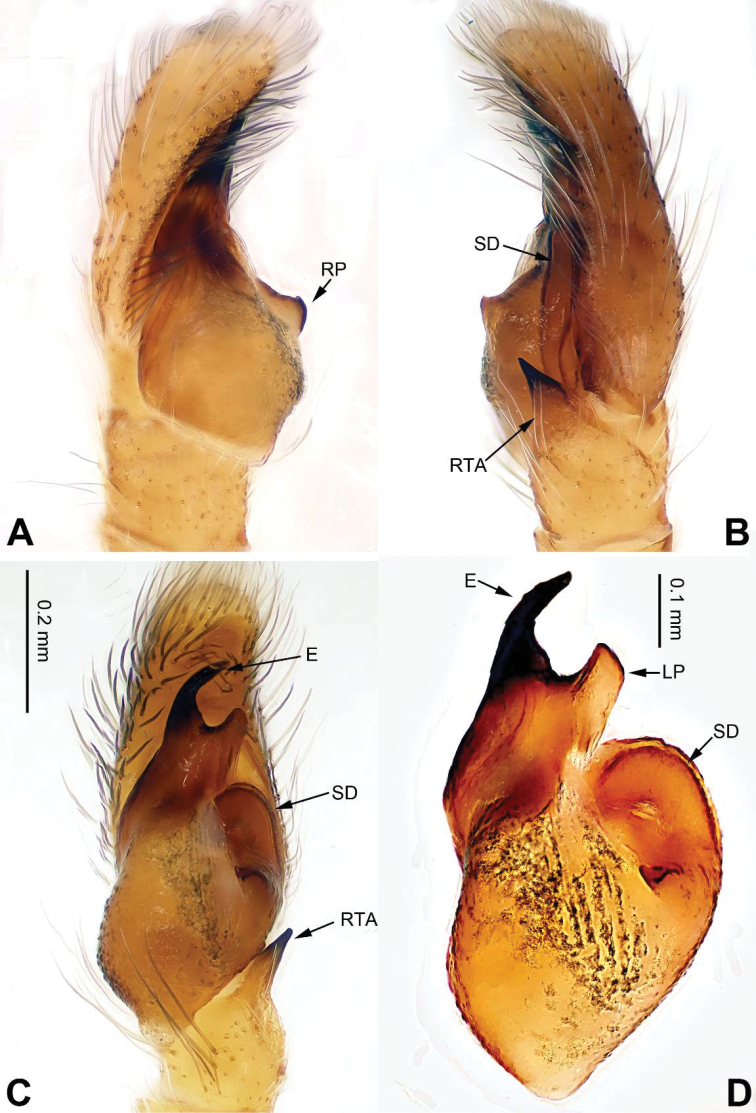
Palp of *Phintella lepidus* sp. n., male holotype. **A** prolateral **B** retrolateral **C** ventral **D** bulb, ventral. Scale bar equal for **A–C**.

**Figure 33.**
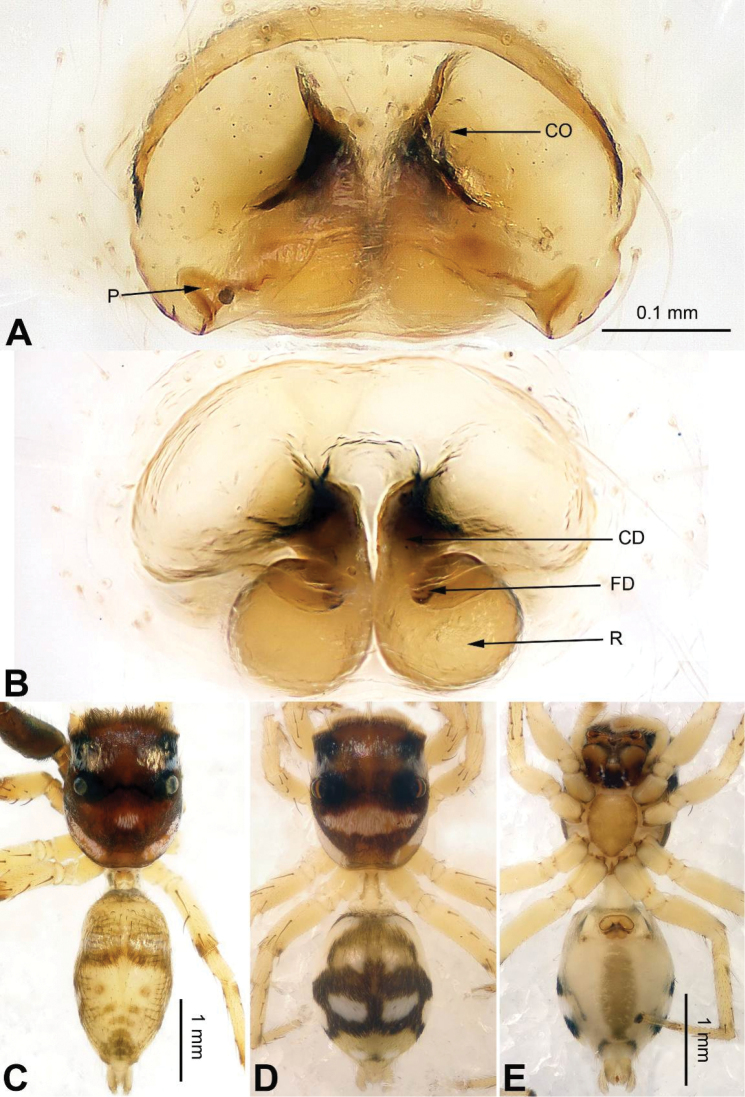
*Phintella lepidus* sp. n., female paratype and male holotype. **A** epigyne, ventral **B** vulva, dorsal **C** male habitus, dorsal **D** female habitus, dorsal **E** female habitus, ventral. Scale bars equal for **A** and **B**; equal for **D** and **E**.

Male can be distinguished from other congeners by the wrench-like structure comprising the embolus and lamellar process (Fig. 32C–D). The epigyne (Fig. 33A–B) resembles that of *Pancorius piatensis* Barrion & Litsinger, 1995 (see Barrion and Litsinger 1995: fig. 36a–f), but the copulatory ducts are broader, about 1/2 the length of the receptacle diameter vs. 1/4 the length in *Pancorius piatensis*.

###### Description.

**Male** (holotype). Total length 5.10, CL 2.25, CW 1.75, AL 2.85, AW 1.50. Eye measurements: AME 0.50, ALE 0.30, PME 0.05, PLE 0.20, AER 1.75, PER 1.70, EFL 1.35. Clypeus 0.40 high. Legs: I 6.25 (1.75, 1.00, 1.50, 1.25, 0.75); II 4.83 (1.35, 0.73, 1.15, 0.90, 0.70); III 5.60(1.60, 0.70, 1.25, 1.30, 0.75); IV 5.80 (1.65, 0.70, 1.35, 1.35, 0.75).

Carapace dark brown (Fig. 33C). Ocular area with metallic lustre, anteriorly with black hairs. Posterior median and margin with white strip of hairs. Clypeus with white strip of hairs. Chelicerae dark brown. *Maxillae and* labium greyish brown, tips with grey hairs. Sternum yellow with dark margin. Atrium with distinct anterior margin. Abdomen oval, dorsomedially yellow, the rest dark grey with a metallic lustre. Venter dark grey. Spinnerets grey-brown. Legs I more robust and darker than others, which are yellowish. Spination of leg I: femur d3-1-1; tibia v2-2-2-2; metatarsus v2-0-2. Palp: tibia short, about 1/3 the length of the cymbium. Tibial apophysis about 2/3 the length of the tibia, with pointed tip (Fig. 32B). Bulb about twice as long than wide, with distinct outgrowth and one retrolateral process (in prolateral or retrolateral views). Embolus subequal to length of the RTA, accompanied with one lamellar process (Fig. 32D).

**Female** (one of paratypes). Total length 4.42, CL 1.70, CW 1.30, AL 2.72, AW 1.72. Eye measurements: AME 0.43, ALE 0.22, PME 0.04, PLE 0.23, AER 1.25, PER 1.23, EFL 1.13. Clypeus 0.37 high. Legs: I 3.35 (0.92, 0.62, 0.91, 0.61, 0.39); II 3.34 (0.90, 0.63, 0.88, 0.56, 0.37); III 3.57(1.06, 0.50, 0.71, 0.86, 0.44); IV 4.15 (1.33, 0.47, 0.96, 0.87, 0.52).

Posterior part of carapace with broader white stripe of hairs than in male. Abdomen with broad, dark decorative pattern. Legs yellowish. Spination of leg I: femur d2-1-1; tibia v2-2-2-2; metatarsus v2-0-2. Other characters similar to these of male. Epigyne sclerotised along the anterior margin (Fig. 33A). Copulatory openings with strongly sclerotised edges. Two pockets near posterolateral edge. Copulatory ducts bent, short. Receptacles spherical, their diameter about 2 times as wide as copulatory ducts. Fertilisation ducts located anteriorly to the receptacles (Fig. 33B).

###### Distribution.

Known from two localities in Xishuangbanna.

##### Phintella sancha

Taxon classificationAnimaliaAraneaeSalticidae

Cao & Li sp. n.

http://zoobank.org/43D9664A-155C-4373-A6E2-7C32C044F02A

Figs 34 , 35 , 43

###### Type.

**Holotype** ♂: CHINA, Yunnan, Mengla County, Menglun Town, rubber plantation (21°54.684'N, 101°16.319'E, 585 m), 8 April 2007, G. Zheng leg.

###### Etymology.

From Chinese Pinyin *san cha* (trident), in reference to the trifurcate RTA; noun.

###### Diagnosis.

**Figure 34.**
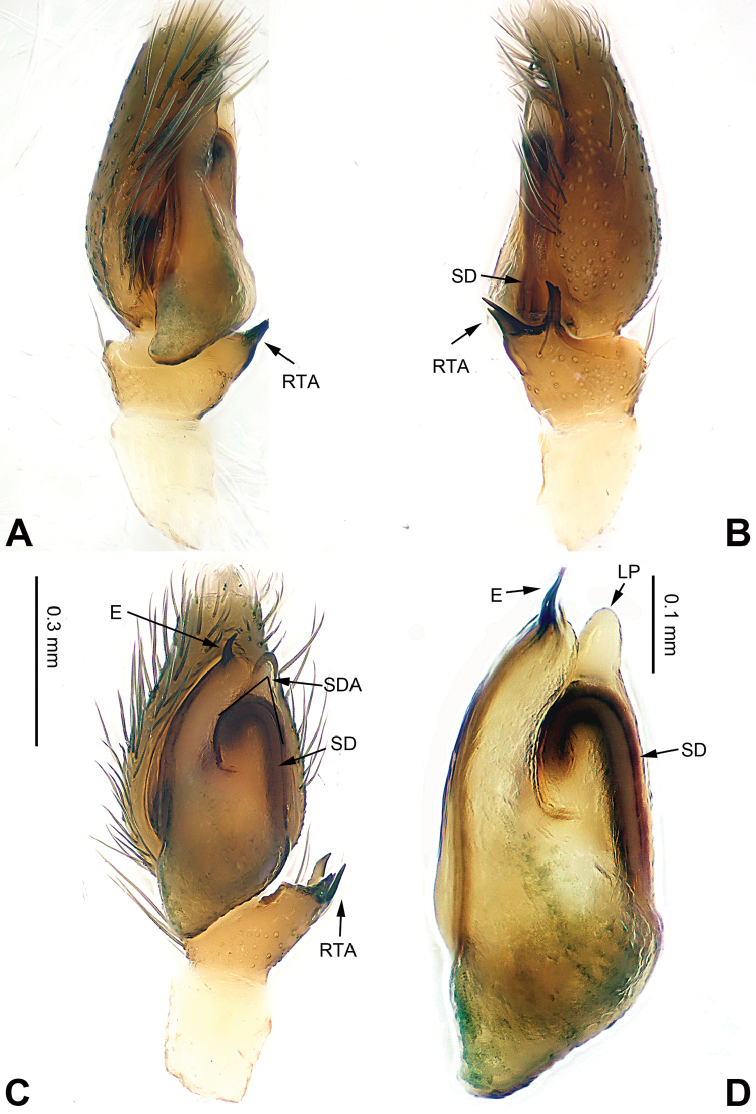
Palp of *Phintella sancha* sp. n., male holotype. **A** prolateral **B** retrolateral **C** ventral **D** bulb, ventral. Scale bar equal for **A–C**.

Similar to *Phintella suavisoides* Lei & Peng, 2013 (Fig. 36), but can be distinguished by: (1) trifurcate RTA in new species (Fig. 34B) vs. bifurcate in *Phintella suavisoides*; (2) the terminal seminal duct angle almost 30° (Fig. 34C) vs. about 60° in *Phintella suavisoides*; (3) the embolus of the new species is accompanied by one digitiform lamellar process (Fig. 34D), lacking in *Phintella suavisoides*.

###### Description.

**Male** (holotype). Total length 3.60, CL 1.75, CW 1.40, AL 1.85, AW 1.09. Eye measurements: AME 0.45, ALE 0.13, PME 0.04, PLE 0.14, AER 1.25, PER 1.20, EFL 1.06. Clypeus 0.15 high. Legs: I 4.63 (1.50, 0.80, 1.13, 0.80, 0.40); II 3.50 (1.05, 0.6, 0.75, 0.65, 0.45); III 4.08 (1.25, 0.55, 0.88, 0.90, 0.50); IV 4.10 (1.05, 0.50, 1.00, 1.00, 0.55).

**Figure 35.**
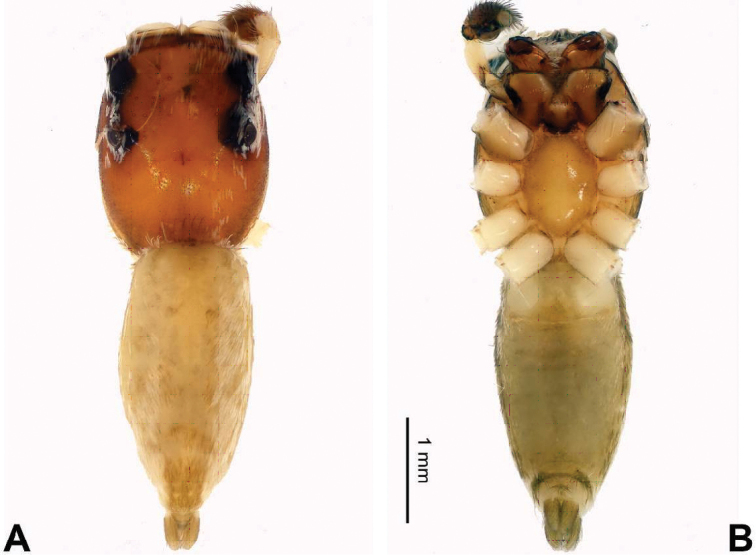
Habitus of *Phintella sancha* sp. n., male holotype. **A** dorsal **B** ventral.

Carapace brown (Fig. 35A). Chelicerae dark brown. *Maxillae* and labium brown, tips white with greyish hairs. Sternum light brown. Abdomen oval, greyish. Venter and spinnerets grey. Legs I more robust and darker than others, which are yellowish. Spination of leg I: femur d2-1-1; tibia v2-2-2; metatarsus v2-0-2. Palp: tibia short, about 1/3 the length of the cymbium. RTA trifurcate (Fig. 34B), short, about 1/4 the length of the cymbium. Bulb 2 times longer than wide, with anterior semi-transparent lobe. Embolus very short, slightly bent (Fig. 34C).

**Female.** Unknown.

###### Distribution.

Known only from the type locality.

##### Phintella suavisoides

Taxon classificationAnimaliaAraneaeSalticidae

Lei & Peng, 2013

Figs 36 , 37 , 43

Phintella suavisoides Lei & Peng, 2013: 103, figs 5, 6a–e (♂).

###### Material examined.

1♂1♀: CHINA, Yunnan, Mengla County, Menglun Town, 48 km landmark in Nature Reserve (21°38.853'N, 101°09.625'E, 1001 m), seasonal rainforest, 30 July 2012, Q. Zhao & Z. Chen leg. 1♂, CHINA, Yunnan, Jinghong City, Menga Town, Wengnan Village (22°05.020'N, 100°22.086'E, 1118 m), secondary forest, 24 July 2012, Q. Zhao & Z. Chen leg.; 1♀, CHINA, Yunnan, Jinghong City, Menghai County, Manda Village (22°01.702'N, 100°23.700'E, 1188 m), secondary forest, 28 July 2012, Q. Zhao & Z. Chen leg.

###### Comparative material examined.

**Holotype** ♂ (Hunan Normal University, China): CHINA, Yunnan, Tengchong County, Jietou Township, Zhoujiapo Village (25°32.086'N, 98°40.139'E, 1620 m), 13 May 2006, C. Yin, X. Peng, J. Hu & P. Hu leg.

###### Diagnosis.

Male well diagnosed by Lei and Peng (2013). The female resembles *Phintella cavaleriei* (Schenkel, 1963) (see Peng et al. 1993: figs 537–539), but the copulatory openings and copulatory ducts located medially (Fig. 37A–B) vs. laterally; copulatory ducts bent dorsally (Fig. 37B) vs. facing each other in *Phintella cavaleriei*.

**Figure 36.**
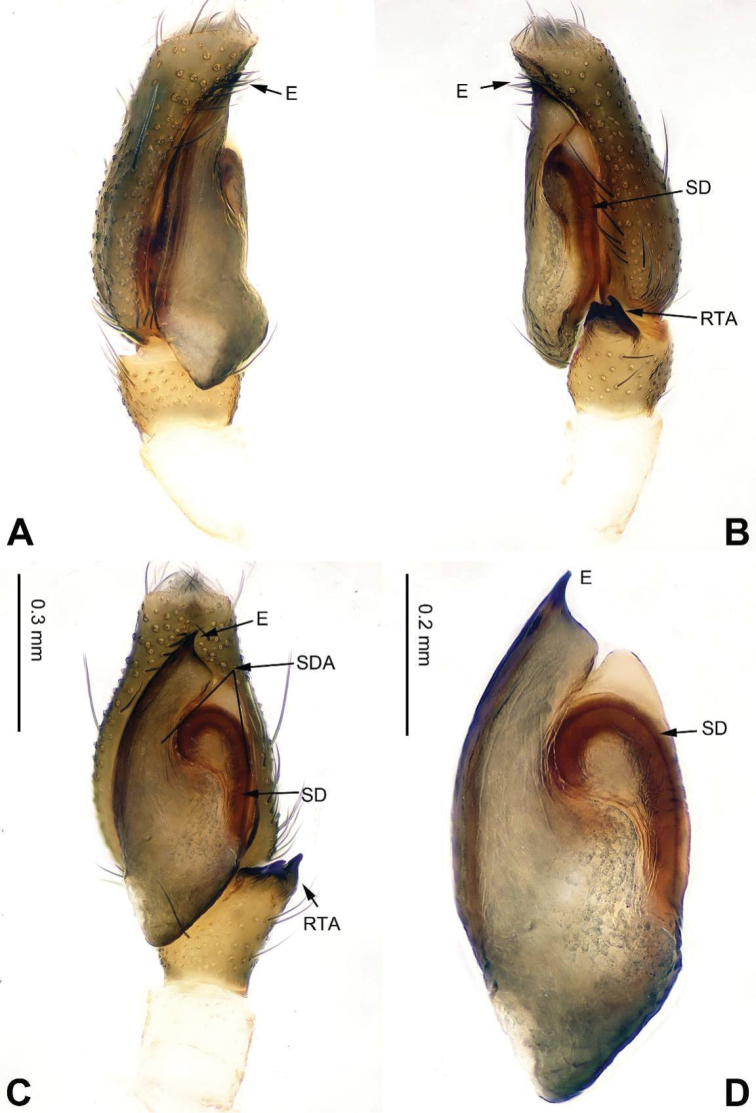
Palp of *Phintella suavisoides*, male from Xishuangbanna. **A** prolateral **B** retrolateral **C** ventral **D** bulb, ventral. Scale bar equal for **A–C**.

**Figure 37.**
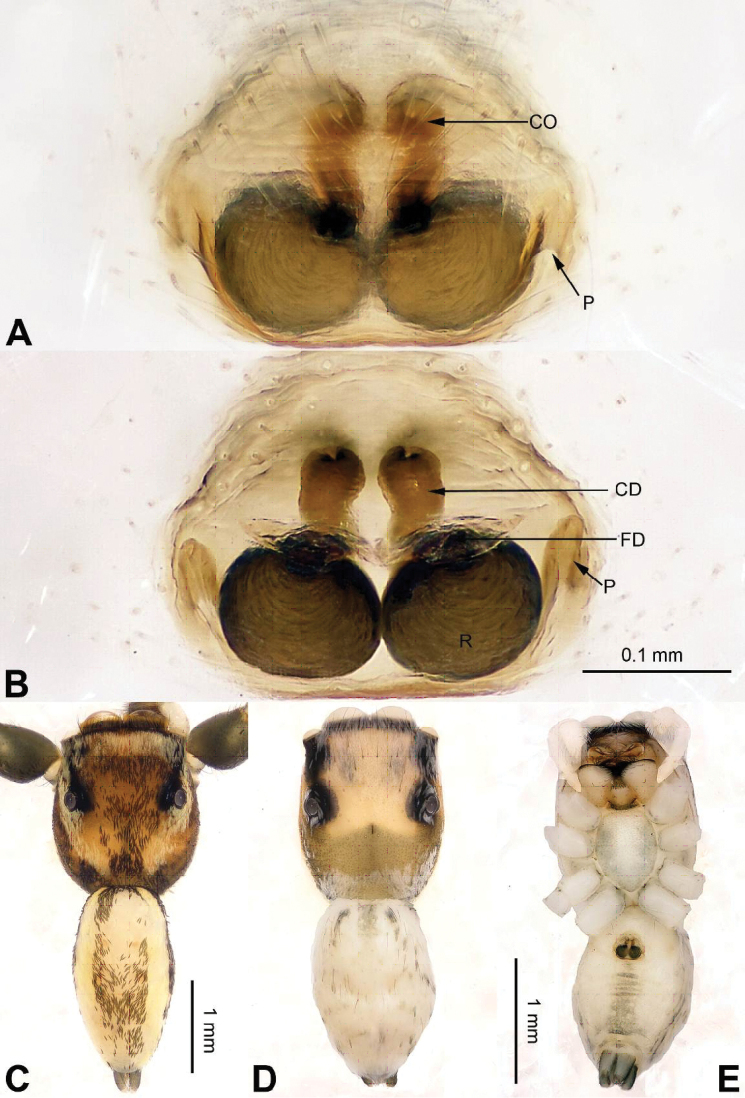
*Phintella suavisoides*, female and male from Xishuangbanna. **A** epigyne, ventral **B** vulva, dorsal **C** male habitus, dorsal **D** female habitus, dorsal **E** female habitus, ventral. Scale bars equal for **A** and **B**; equal for **D** and **E**.

###### Description.

**Male.** Well described by Lei and Peng (2013).

**Female.** Total length 3.25, CL 1.50, CW 1.28, AL 1.75, AW 1.10. Eye measurements: AME 0.38, ALE 0.20, PME 0.03, PLE 0.15, AER 1.19, PER 1.20, EFL 1.00. Clypeus 0.10 high. Legs: I 3.01 (0.98, 0.50, 0.63, 0.50, 0.40); II 2.62 (0.80, 0.40, 0.52, 0.50, 0.40); III 3.09 (1.00, 0.40, 0.56, 0.73, 0.40); IV 3.73 (1.18, 0.44, 0.85, 0.86, 0.40).

Carapace light grey with dense setae (Fig. 37C). Clypeus light brown, covered by white, flat hairs. Chelicerae brown. Maxillae and labium greyish, tips with black hairs. Sternum greyish brown with light margin. Abdomen oval and white, clothed in dense setae. Venter greyish. Spinnerets green-grey. Legs white. Spination of leg I: femur d0-1-0; tibia v2-2-2; metatarsus v2-0-2. Copulatory openings small and located anteromedially (Fig. 37A). Copulatory ducts bent, short. Receptacles spherical, diameters four times wider than copulatory ducts. Fertilisation ducts located anteriorly to receptacles (Fig. 37B).

###### Distribution.

Known from Gaoligong Mountains and Xishuangbanna in Yunnan, China.

#### Genus *Ptocasius* Simon, 1885

##### Ptocasius paraweyersi

Taxon classificationAnimaliaAraneaeSalticidae

Cao & Li sp. n.

http://zoobank.org/641FCCBC-0024-43AC-AE43-AE7FADBF0EB4

Figs 38 , 39 , 43

###### Type.

**Holotype** ♂: CHINA, Yunnan, Mengla County, Menglun Town, 48 km landmark in Nature Reserve (21°58.704'N, 101°19.748'E, 1088 m), seasonal rainforest, 12 August 2011, Q. Zhao & Z. Chen leg. **Paratypes**: 1♂2♀, CHINA, Yunnan, Mengla County, Xiaolongha Village, Xishuangbanna Nature Reserve, Biological diversity corridor (21°24.192'N, 101°37.025'E, 657 m), seasonal rainforest, 29 June 2012, Q. Zhao & Z. Chen leg.

###### Etymology.

From Greek prefix *para* and *weyersi*, a patronym from Weyers, referring to similarities with *Ptocasius weyersi*.

###### Diagnosis.

**Figure 38.**
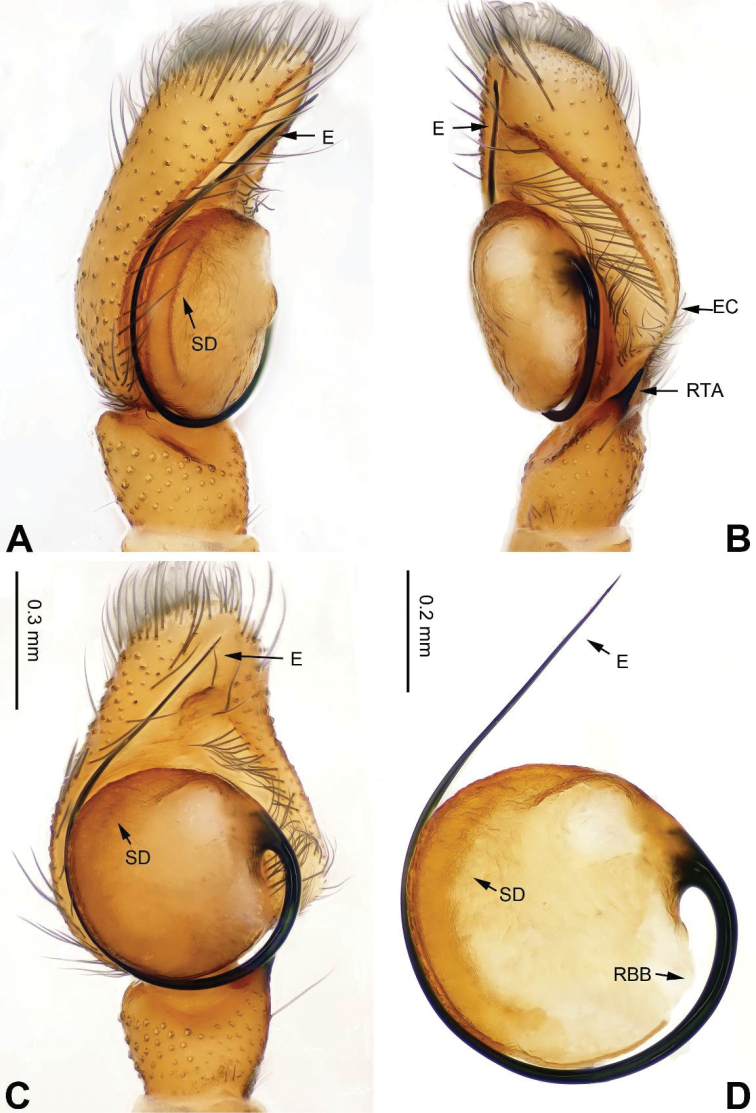
Palp of *Ptocasius paraweyersi* sp. n., male holotype. **A** prolateral **B** retrolateral **C** ventral **D** bulb, ventral. Scale bar equal for **A–C**.

**Figure 39.**
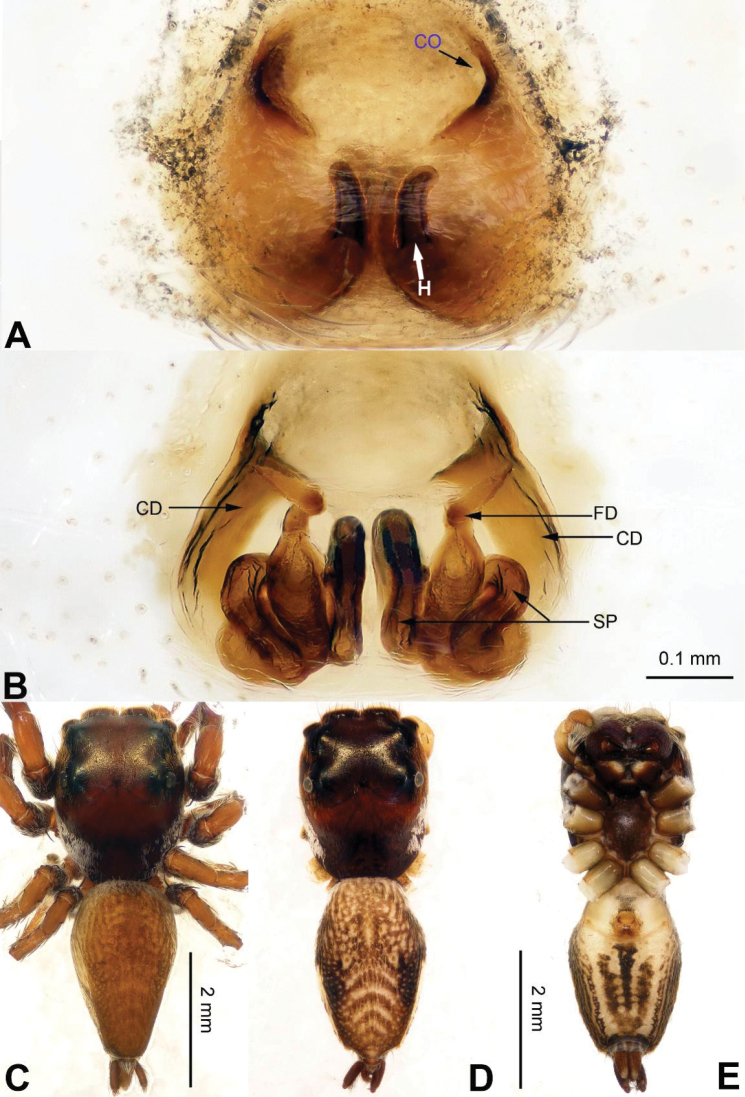
*Ptocasius paraweyersi* sp. n., female paratype and male holotype. **A** epigyne, ventral **B** vulva, dorsal **C** male habitus, dorsal **D** female habitus, dorsal **E** female habitus, ventral. Scale bars equal for **A** and **B**; equal for **D** and **E**.

Similar to *Ptocasius weyersi* (see Żabka 1985: figs 530–532), but the tegulum has one small bump (Fig. 38D) and the RTA is straight (Fig. 38B) vs. bent backward in *Ptocasius weyersi*. The female resembles *Ptocasius weyersi*, ﻿but differs by having two epigynal hoods (Fig. 39A) vs. one. Compared to *Ptocasius songi* Logunov, 1995, the hoods are located medially (Fig. 39A) vs. laterally and the receptacles are elongate (Fig. 39B) vs. spherical.

###### Description.

**Male** (holotype). Total length 6.10, CL 2.60, CW 2.00, AL 3.50, AW 1.65. Eye measurements: AME 0.63, ALE 0.31, PME 0.05, PLE 0.30, AER 1.90, PER 1.90, EFL 1.60. Clypeus 0.15 high. Legs: I 5.20 (1.50, 1.00, 1.25, 0.85, 0.60); II 4.45(1.35, 0.75, 1.10, 0.70, 0.55); III 5.15 (1.55, 0.75, 1.10, 1.05, 0.70); IV 5.25 (1.55, 0.75, 1.10, 1.10, 0.75).

Carapace dark brown with dense white hairs on both sides of the posterior edge (Fig. 39C). Chelicerae, clypeus and labium dark brown. *Maxillae* brown with wide, white tips. Sternum dark brown. Abdomen elongate, brownish. Venter puce with longitudinal rows of dots. Legs dark brown. Spination of leg I: femur d5-1-1; patella p0-1-0, r0-1-0; tibia v2-2-2, p1-1-1, r1-1-1; metatarsus v2-0-2, p1-0-1, r1-0-1. Palp: tibia short, about 1/3 the length of the cymbium. RTA short, with a pointed tip (Fig. 38C). Cymbium nearly flabellate with retrolateral fold. Seminal duct encircling tegulum prolaterally. Embolus elongate, its base at two o’clock (Fig. 38C–D).

**Female** (one of paratypes). Total length 6.25, CL 2.81, CW 2.03, AL 3.44, AW 1.85. Eye measurements: AME 0.63, ALE 0.33, PME 0.05, PLE 0.28, AER 1.98, PER 2.00, EFL 1.72. Clypeus 0.10 high. Legs: I 4.57 (1.41, 1.00, 1.00, 0.63, 0.53); II 4.37 (1.41, 0.90, 0.93, 0.63, 0.50); III 4.80 (1.56, 0.76, 1.00, 0.92, 0.56); IV 5.37 (1.70, 0.80, 1.13, 1.10, 0.64).

Abdomen light brown with irregular white patches. Venter yellowish with black longitudinal stripe. Spination of leg I: femur d3-1-1; patella p0-1-0; tibia v2-2-2, p1-0-1; metatarsus v2-0-2. Other characters similar to the male. Copulatory ducts long, broad and located laterally. Receptacles long and convoluted, forming four loops. Fertilisation ducts located at the anterior part of the receptacles (Fig. 39B).

###### Distribution.

Known from several localities in Xishuangbanna.

#### Genus *Stenaelurillus* Simon, 1886

##### Stenaelurillus fuscus

Taxon classificationAnimaliaAraneaeSalticidae

Cao & Li sp. n.

http://zoobank.org/33962F70-D32A-46C9-8684-83B8E7228F07

**Figure 40.**
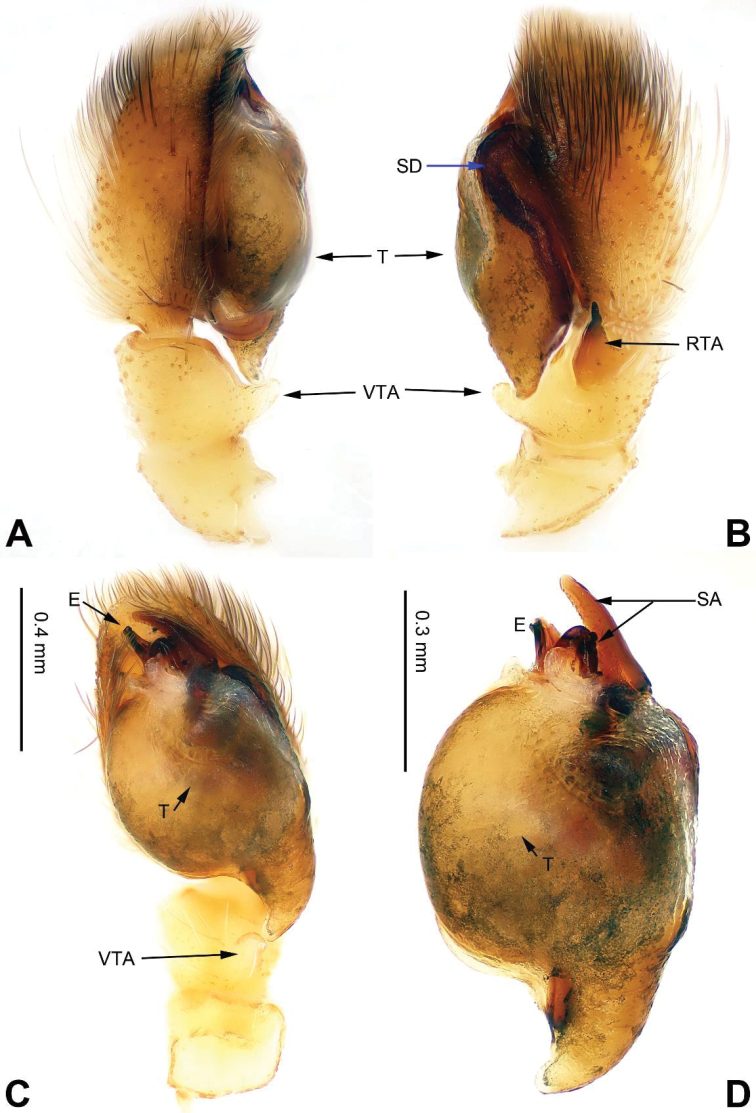
Palp of *Stenaelurillus fuscus* sp. n., male holotype. **A** prolateral **B** retrolateral **C** ventral **D** bulb, ventral. Scale bar equal for **A–C**.

**Figure 41.**
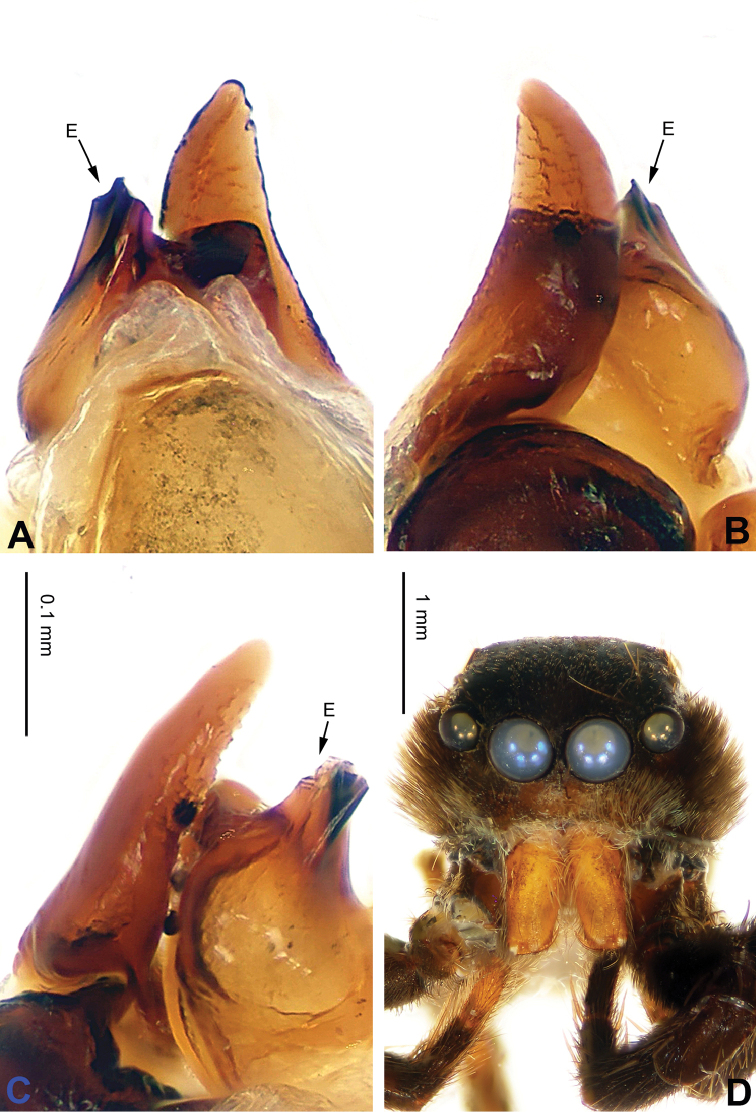
*Stenaelurillus fuscus* sp. n., male holotype. **A** embolus, prolateral **B** embolus, retrolateral **C** embolus, dorsal **D** habitus, front. Scale bars equal for **A–C**.

**Figure 42.**
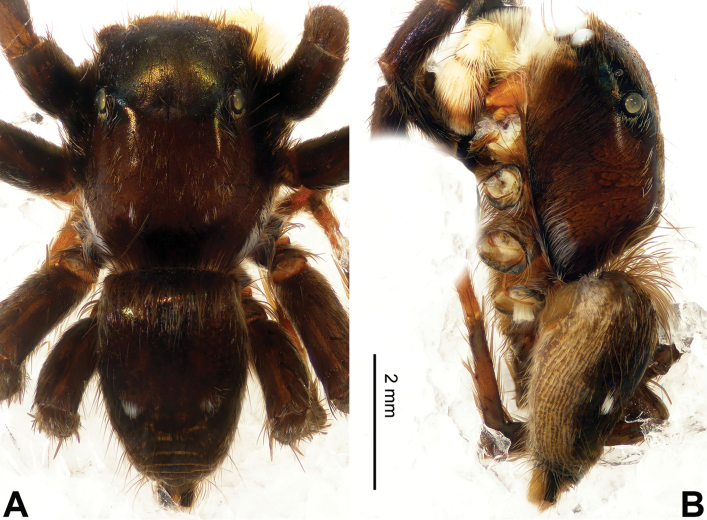
Habitus of *Stenaelurillus fuscus* sp. n., male holotype. **A** dorsal **B** lateral.

**Figure 43.**
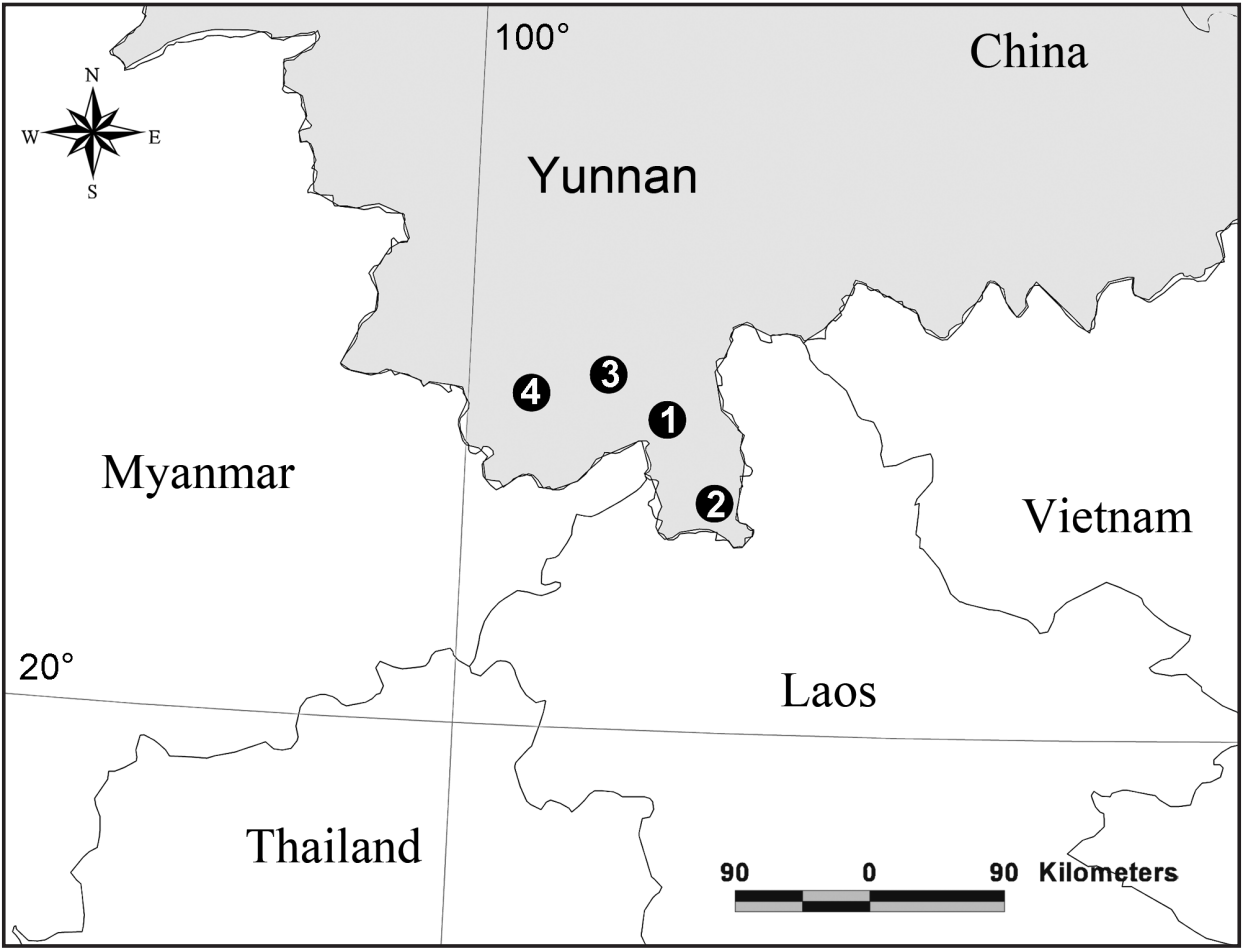
Four main collection localities in Xishuangbanna, Yunnan, China. **1** Mengla Town **2** Menglun Town **3** Mengyang Town **4** Menghai Town.

Figs 40 , 41 , 42 , 43

###### Type.

**Holotype** ♂: CHINA, Yunnan, Mengla County, Menglun Town, Rubber-Tea plantation (21°55.551'N, 101°16.923'E, 561 m), 11 December 2006, G. Zheng leg.

###### Etymology.

From Latin *fuscus* (dark), in reference to the dark carapace; adjective.

###### Diagnosis.

Similar to *Stenaelurillus minutus* Song & Chai, 1991 (see Wesołowska 2014: fig. 4A–D), but embolus straight (Fig. 40C) vs. bent; sclerotized apophysis (the longer one) located anteriorly to embolus (Fig. 40C) vs. posteriorly; RTA almost triangular (Fig. 40B) vs. broad with thin, long, pointed apex in *Stenaelurillus minutus*.

###### Description.

**Male** (holotype). Total length 5.30, CL 2.80, CW 2.35, AL 2.50, AW 1.60. Eye measurements: AME 0.48, ALE 0.30, PME 0.06, PLE 0.29, AER 1.75, PER 1.60, EFL 1.30. Clypeus height 0.31 high. Legs: I 4.50 (1.50, 0.75, 1.00, 0.75, 0.50); II 6.85 (1.50, 0.65, 0.95, 0.70, 0.50); III 5.35 (1.80, 0.70, 1.15, 1.15, 0.55); IV 5.85 (2.00, 0.75, 1.25, 1.25, 0.60).

Carapace dark, moderately high and slightly broadened posteriorly (Fig. 42A). Lateral carapace margins with long, dense brush-like setae. Chelicerae dark brown. *Maxillae* and labium brown, tips light with greyish hairs. Sternum dark, oval. Abdomen shield-shaped, anterior edge with long dense bristles. Venter and spinnerets dark grey. Legs dark with dense hairs and numerous spines. Palpal tibia white and short, about 1/3 the length of the cymbium. Tibia with ventral, digitiform, obtuse apophysis and triangular RTA (Fig. 40B). Seminal duct encircling retrolateral part of tegulum. Tegulum oval, with long and triangular posterior lobe.

**Female.** Unknown.

###### Distribution.

Known only from the type locality.

## Supplementary Material

XML Treatment for Afraflacilla ballarini

XML Treatment for Agorius tortilis

XML Treatment for Bavia capistrata

XML Treatment for Bavia exilis

XML Treatment for Carrhotus kevinlii

XML Treatment for Carrhotus sarahcrewsae

XML Treatment for Chinattus wengnanensis

XML Treatment for Chinophrys mengyangensis

XML Treatment for Cocalus menglaensis

XML Treatment for Cosmophasis xiaolonghaensis

XML Treatment for Cytaea yunnanensis

XML Treatment for Gedea pinguis

XML Treatment for Gelotia zhengi

XML Treatment for Icius bamboo

XML Treatment for Nannenus menghaiensis

XML Treatment for Pancorius latus

XML Treatment for Phintella lepidus

XML Treatment for Phintella sancha

XML Treatment for Phintella suavisoides

XML Treatment for Ptocasius paraweyersi

XML Treatment for Stenaelurillus fuscus

## References

[B1] Andreeva EM Hęciak S Prószyński J (1984) Remarks on *Icius* and *Pseudicius* (Araneae, Salticidae) mainly from central Asia. Annales Zoologici, Warszawa 37: 349–375.

[B2] Barrion AT Litsinger JA (1995) Riceland Spiders of South and Southeast Asia. CAB International, Wallingford, 700 pp.

[B3] Folmer O Black M Hoeh W Lutz R Vrijenhoek R (1994) DNA primers for amplification of mitochondrial cytochrome c oxidase subunit I from diverse metazoan invertebrates. Molecular Marine Biology and Biotechnology 3(5): 294–299. 7881515

[B4] Koch CL (1846) Die Arachniden. Nürnberg, Dreizehnter Band, 234 pp [Vierzehnter Band, 88 pp]

[B5] Lei H Peng XJ (2013) Five new species of the genus *Phintella* (Araneae: Salticidae) from China. Oriental Insects 47: 99–110. doi: 10.1080/00305316.2013.783747

[B6] Li S Lin Y (2016) Species Catalogue of China. Volume 2. Animals. Invertebrates (I), Arachnida: Araneae. Science Press, Beijing, 549 pp.

[B7] Miller JA Carmichael A Ramirez MJ Spagna JC Haddad CR Řezáč M Johannesen J Král J Wang XP Griswold CE (2010) Phylogeny of entelegyne spiders: affinities of the family Penestomidae (new rank), generic phylogeny of Eresidae, and asymmetric rates of change in spinning organ evolution (Araneae, Araneoidea, Entelegynae). Molecular Phylogenetics and Evolution 55: 786–804. doi: 10.1016/j.ympev.2010.02.021 20206276 10.1016/j.ympev.2010.02.021

[B8] Ono H Thinh TH Pham DS (2012) Spiders (Arachnida, Araneae) recorded from Vietnam, 1837–2011. Memoirs of the National Museum of Nature and Science, Tokyo 48: 1–37.

[B9] Peng XJ Xie LP Xiao XQ Yin CM (1993) Salticids in China (Arachniuda: Araneae). Hunan Normal University Press, 270 pp.

[B10] Platnick NI Shadab MU (1975) A revision of the spider genus *Gnaphosa* (Araneae, Gnaphosidae) in America. Bulletin of the American Museum of Natural History 155: 1–66.

[B11] Prószyński J (1992) Salticidae (Araneae) of the Old World and Pacific Islands in several US collections. Annales Zoologici, Warszawa 44: 87–163.

[B12] Prószyński J (2009) Comments on the Oriental genera *Agorius* and *Synagelides* (Araneae: Salticidae). In: Makarov SE Dimitrijević RN (Eds) Advances in Arachnology and Developmental Biology. Institute of Zoology, Bulgarian Academy of Sciences Monographs 12: 311–325.

[B13] Prószyński J Deeleman-Reinhold CL (2012) Description of some Salticidae (Aranei) from the Malay archipelago. II. Salticidae of Java and Sumatra, with comments on related species. Arthropoda Selecta 21: 29–60.

[B14] Wanless FR (1981) A revision of the spider genus *Cocalus* (Araneae: Salticidae). Bulletin of the British Museum of Natural History (Zool.) 41: 253–261.

[B15] Wesołowska W (2014) A review of the Asian species of the spider genus *Stenaelurillus* (Araneae: Salticidae). Oriental Insects 47(4): 246–254. doi: 10.1080/00305316.2013.871823

[B16] World Spider Catalog (2016) World Spider Catalog. Natural History Museum Bern http://wsc.nmbe.ch [version 17.0, accessed 2 October, 2016]

[B17] Yang YT Tang YQ (1997) Two new species of the family Salticidae from China (Araneae). Journal of Lanzhou University (Natural Sciences) 33: 93–96.

[B18] Żabka M (1985) Systematic and zoogeographic study on the family Salticidae (Araneae) from Viet-Nam. Annales Zoologici, Warszawa 39: 197–485.

[B19] Żabka M (1988) Salticidae (Araneae) of Oriental, Australian and Pacific regions, III. Annales Zoologici, Warszawa 41: 421–479.

[B20] Żabka M (1993) Salticidae (Arachnida: Araneae) of the Oriental, Australian and Pacific regions. IX. Genera *Afraflacilla* Berland & Millot 1941 and *Evarcha* Simon 1902. Invertebrate Taxonomy 7: 279–295. doi: 10.1071/IT9930279

[B21] Żabka M (1997) Salticidae: Pająki skaczące (Arachnida: Araneae). Fauna Polski 19: 1–188.

[B22] Żabka M Waldock J (2012) Salticidae (Arachnida: Araneae) from Oriental, Australian and Pacific regions. Genus *Cosmophasis* Simon, 1901. Annales Zoologici, Warszawa 62: 115–198. doi: 10.3161/000345412X633694

[B23] Zhang JX Maddison WP (2012) New euophryine jumping spiders from Southeast Asia and Africa (Araneae: Salticidae: Euophryinae). Zootaxa 3581: 53–80.

[B24] Zheng G Li S Yang X (2015) Spider diversity in canopies of Xishuangbanna rainforest (China) indicates an alarming juggernaut effect of rubber plantations. Forest Ecology and Management 338: 200–207. doi: 10.1016/j.foreco.2014.11.031

[B25] Zhu H Xu ZF Wang H Li BG (2004) Tropical rainforest fragmentation and its ecological and species diversity changes in southern Yunnan. Biodiversity and Conservation 13(7): 1355–1372. doi: 10.1023/B:BIOC.0000019397.98407.c3

